# Anti-Inflammatory Effect of Palmatine Chloride on Lipopolysaccharide-Stimulated RAW 264.7 Mouse Macrophages via Calcium-CHOP Pathway

**DOI:** 10.3390/ijms27135704

**Published:** 2026-06-24

**Authors:** Young-Jin Kim, Wansu Park

**Affiliations:** Department of Pathology, College of Korean Medicine, Gachon University, Seongnam 13120, Republic of Korea

**Keywords:** palmatine chloride, lipopolysaccharide, macrophage, CHOP, cytokine, p38 MAPK, endoplasmic reticulum, nitric oxide, cytosolic calcium

## Abstract

Palmatine chloride (berbericinine, C_21_H_22_ClNO_4_) is a protoberberine alkaloid found in several plants, including Rhizoma Coptidis, Cortex Phellodendri, Rhizoma Corydalis, Guduchi (*Tinospora cordifolia*), and *Tinospora sagittata* roots. Palmatine chloride (PA) is known as an inhibitor of dopamine generation. However, its effect on endoplasmic reticulum (ER) stress-related macrophage activation caused by endotoxin (lipopolysaccharide) is not yet well known. In this study, the effects of PA on pyroptotic responses of mouse macrophages (RAW 264.7) activated by endotoxin were investigated using Griess reagent assay for nitric oxide (NO) production, fluo-4 assay for cytosolic calcium release, dihydrorhodamine 123 assay for hydrogen peroxide production, multiple cytokine assay for cytokine production, real-time PCR for inflammatory gene transcriptions, and flow cytometry assay for p38 MAPK activation. Preliminary experiments using THP-1 human monocytic cells demonstrated that PA was not cytotoxic and significantly reduced basal NO production. Results revealed that PA significantly reduced excessive production levels of NO, hydrogen peroxide, pro-inflammatory cytokines (such as interleukin (IL)-6, CCL3 (MIP-1α), and CSF2 (GM-CSF)), and cytosolic calcium release in endotoxin-stimulated RAW 264.7, but significantly increased the production of anti-inflammatory cytokine IL-10. PA inhibited endotoxin-induced transcripts of *Chop*, *Stat1*, *Fas*, and *c-Fos* in activated RAW 264.7. It also decreased p38 MAPK phosphorylation and level of Fas in RAW 264.7 stimulated by endotoxin. To further interpret these findings, a network pharmacology-informed analysis based on large-scale literature mining was performed, supporting the multi-target regulatory role of PA in ER stress-related pathways. Briefly, PA exerts anti-inflammatory effects on endotoxin-stimulated RAW 264.7 via the calcium-CHOP pathway, consequently reducing endotoxin-induced production of pro-inflammatory mediators (NO, cytokines, etc.) and relieving ER stress-related pyroptotic cascade.

## 1. Introduction

Infection caused by bacterial invasion to the human body still threatens human health, although many powerful antibiotics have been developed [1,2]. Therefore, research on natural products with fewer side effects for the development of treatments for bacterial infection continues [3,4]. Many studies have shown that natural products exhibit anti-inflammatory effects by controlling macrophage activation triggered by infectious pathogens [5,6,7]. To treat infectious diseases, direct removal of infectious pathogens is important. Regulating excessive inflammatory responses caused by infection is also meaningful. Lipopolysaccharides (LPS) are endotoxins found in the outer membrane of Gram-negative bacteria [8,9]. Because macrophages stimulated by endotoxin release large amounts of cytokines and nitrogen oxides (NO), it is meaningful to perform research on natural products that can regulate LPS-induced production of inflammatory mediators in macrophages.

Macrophage activation is related to many inflammatory diseases. Many studies have been conducted on endoplasmic reticulum (ER) stress and transcription factor C/EBP homologous protein (CHOP) pathway related to macrophage activation caused by various stimuli such as bacterial infection or free cholesterol. Our previous study has shown that baicalin can inhibit the inflammatory response of RAW 264.7 mouse macrophages stimulated by LPS through the calcium–CHOP pathway involved in the ER stress cascade [7]. In other words, ER stress is an important mechanism involved in the activation of macrophages caused by endotoxins. ER stress induced by free cholesterol or infectious stimuli has been implicated in macrophage activation and inflammatory apoptosis through calcium dysregulation [10,11,12,13,14,15,16,17]. LPS might activate macrophages to produce various cytokines through p38 mitogen-activated protein kinase (MAPK) signaling [18]. Previous studies have suggested that p38 MAPK-mediated CHOP activation contributes to ER stress-associated inflammatory responses and cytokine production [19,20,21]. Previous studies have suggested that excessive NO and ROS production may contribute to ER stress, calcium dysregulation, and inflammatory cell death in activated macrophages [22,23,24,25,26,27,28,29,30,31]. Tabas et al. [10] have reported that macrophages with intensified ER stress undergo apoptosis by increasing calcium release from the ER due to the activation of CHOP, followed by CAMKII-induced ROS production, STAT1 activation, and Fas death receptor (Fas) expression. Seimon et al. [32] have reported that LPS could activate Toll-like receptor 4 (TLR4) in ER-stressed macrophages, in which cytosolic calcium plays a key role. Furthermore, endotoxin-stimulated macrophages can undergo inflammatory programmed cell death associated with ER stress-related signaling pathways and the release of pro-inflammatory mediators [33,34,35]. Activator protein 1 (AP-1) is known to be activated in response to various cell stimuli, including cytokines, oxidative stress, and infections via p38 MAPK signaling, consequently triggering the CHOP pathway [36,37,38]. Interestingly, Klymenko et al. [12] have reported that AP-1-induced CHOP, an important ER stress transcription factor, can increase the release of calcium from ER stores of macrophages stimulated by infectious pathogens, resulting in apoptosis due to ER stress, which can increase the expression of Fas and activate STAT1.

**Figure 1:**
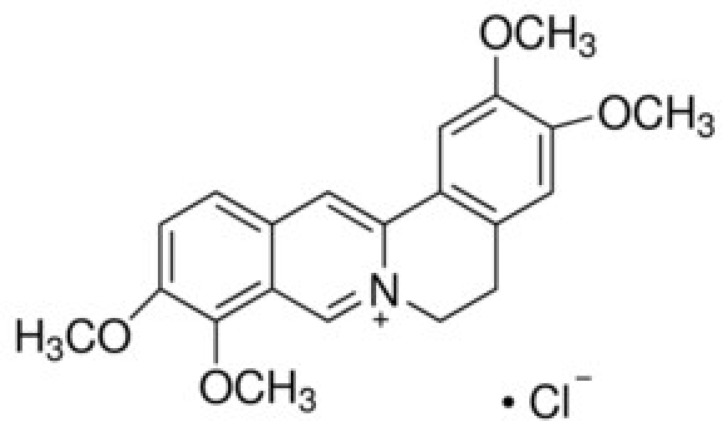
Chemical structure of palmatine chloride (PA).

In a previous study, we reported that berberine can modulate macrophage activation [39]. Previous studies have reported that palmatine chloride (Figure 1) possesses anti-inflammatory, antimicrobial, antioxidant, neuroprotective, hepatoprotective, and metabolic regulatory activities through modulation of multiple signaling pathways [40,41,42,43,44,45,46]. Despite these findings, the effects of PA on endoplasmic reticulum (ER) stress-associated macrophage activation and calcium–CHOP signaling pathways induced by endotoxin stimulation remain insufficiently characterized.

The present study aimed to investigate the effects of PA on LPS-induced macrophage activation, with a particular focus on ER stress and calcium signaling. Furthermore, to extend mechanistic insights beyond experimental observations, a network pharmacology-informed approach based on large-scale literature mining was integrated, enabling a more comprehensive characterization of PA as a multi-target regulator of ER stress, particularly via the calcium–CHOP signaling axis.

## 2. Results

### 2.1. Cell Viability

**Figure 2:**
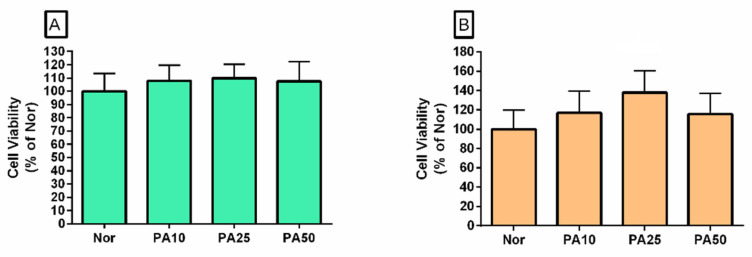
Effects of palmatine chloride on cell viability of THP-1 cells (**A**) and RAW 264.7 cells (**B**). Values are presented as mean ± standard deviation. Nor was the group treated with media alone. PA10 means 10 μM of palmatine chloride, PA25 means 25 μM of palmatine chloride, and PA50 means 50 μM of palmatine chloride.

Viabilities of THP-1 incubated with PA at concentrations of 10, 25, and 50 μM were 107.82 ± 11.86%, 109.92 ± 10.36%, and 107.49 ± 14.68%, respectively, of the normal group (Nor) treated with media only (Figure 2A). Viabilities of RAW 264.7 cells incubated with PA at concentrations of 10, 25, and 50 μM were 116.87 ± 22.49%, 137.96 ± 122.38%, and 115.60 ± 21.38% of the normal group (Nor), respectively (Figure 2B). No cytotoxicity was observed in RAW 264.7 cells treated with PA at concentrations up to 50 μM.

### 2.2. NO Production

**Figure 3:**
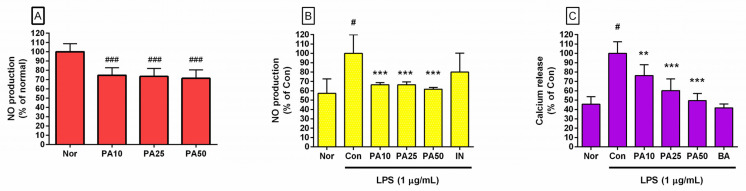
Effects of palmatine chloride on Nitric Oxide (NO) production in unstimulated THP-1 cells (**A**), NO production in lipopolysaccharide (LPS)-stimulated RAW 264.7 cells (**B**), and calcium release in LPS-stimulated RAW 264.7 cells (**C**). Values are presented as mean ± standard deviation. Nor was the group treated with media alone; Con was the group treated with LPS alone. PA10 means 10 μM of palmatine chloride, PA25 means 25 μM of palmatine chloride, and PA50 means 50 μM of palmatine chloride. IN, indomethacin (0.5 µM); BA, baicalein (25 µM). ^#^, *p* < 0.05 vs. Nor; ^###^, *p* < 0.001 vs. Nor; **, *p* < 0.01 vs. Con; ***, *p* < 0.001 vs. Con.

Palmatine chloride significantly reduced basal NO production in THP-1 cells (Figure 3A). In addition, palmatine chloride significantly reduced LPS-induced NO production in RAW 264.7 cells (Figure 3B). In detail, NO production levels in THP-1 cells treated with palmatine chloride at concentrations of 10, 25, and 50 μM were 74.65 ± 8.24%, 73.35 ± 8.69%, and 71.49 ± 8.96%, respectively, of Nor (treated with media only). NO production levels in LPS-stimulated RAW 264.7 cells treated with palmatine chloride at concentrations of 10, 25, and 50 μM were 66.59 ± 2.17%, 66.54 ± 2.99%, and 61.77 ± 1.91%, respectively, of the control group (Con) treated with LPS (1 µg/mL) alone.

These results suggest that palmatine chloride can reduce the level of NO overproduced in activated macrophages in a concentration-dependent manner. NO, as a free radical, is one of the RNS. Although NO can act as a vasorelaxant and neurotransmitter, it can cause pathological conditions, such as damaging surrounding tissues, when it is excessively produced from immune cells, such as macrophages, during infection-induced excessive inflammatory reactions [22,23,24]. NO can also cause ER stress in cells such as pancreatic beta cells and macrophages, inducing intracellular calcium release from the ER stores and promoting CHOP activation with an increase in cytosolic calcium, resulting in cell apoptosis and tissue damage [25,26,27,28]. Therefore, the action of palmatine chloride, which inhibits the overproduction of NO in excited macrophages, might modulate ER stress and inflammation related to endotoxin-stimulated macrophages. Indomethacin (0.5 μM) and baicalein (25 μM) were included as reference compounds for comparison with PA.

### 2.3. Cytosolic Calcium Release

Palmatine chloride significantly reduced the endotoxin-induced release of calcium from ER stores in LPS-stimulated RAW 264.7 (Figure 3C). Cytosolic calcium levels in endotoxin-stimulated RAW 264.7 treated with palmatine chloride at concentrations of 10, 25, and 50 μM were 76.21 ± 11.76%, 60.24 ± 12.46%, and 49.46 ± 7.63% of Con, respectively. These results show that palmatine chloride can decrease cytosolic calcium levels in activated macrophages in a dose-dependent manner. Increased cytosolic calcium in macrophages, excited by stimulating factors such as infectious pathogens, is linked to ER stress amplification and induction of the CHOP pathway in activated macrophages, leading to a programmed inflammatory cell lysis due to increased production of inflammatory factors such as cytokines and increased expression of death receptor Fas [10,26,27,28]. These data suggest that the action of palmatine chloride, which reduces cytosolic calcium in activated macrophages, can alleviate the cascade of apoptosis in macrophages caused by pathogen stimulation by inhibiting the ER stress-related calcium pathway.

### 2.4. Hydrogen Peroxide Production

**Figure 4:**
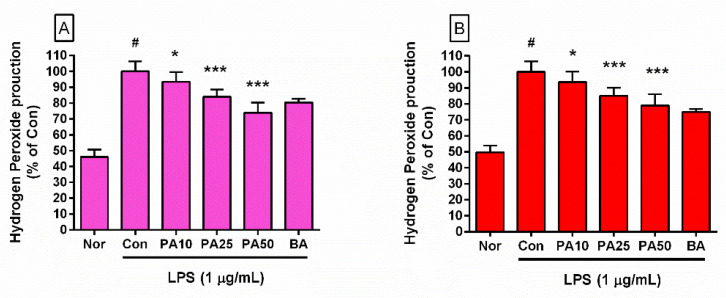
Effects of palmatine chloride on hydrogen peroxide production in lipopolysaccharide (LPS)-stimulated RAW 264.7 macrophages after treatment for 24 h (**A**) and 48 h (**B**). Values are presented as mean ± standard deviation. Nor, the group treated with media alone; Con, the group treated with LPS alone. PA10 means 10 μM of palmatine chloride, PA25 means 25 μM of palmatine chloride, and PA50 means 50 μM of palmatine chloride. BA, baicalein (25 µM). ^#^, *p* < 0.05 vs. Nor; *, *p* < 0.05 vs. Con; ***, *p* < 0.001 vs. Con.

PA significantly reduced endotoxin-induced production of hydrogen peroxide in LPS-stimulated RAW 264.7 cells (Figure 4). Production levels of hydrogen peroxide in RAW 264.7 treated for 24 h with palmatine chloride at concentrations of 10, 25, and 50 μM were 93.42 ± 6.1%, 83.85 ± 4.62%, and 73.93 ± 6.29% of Con, respectively (Figure 4A). After treatment for 48 h, production levels of hydrogen peroxide in RAW 264.7 cells treated with palmatine chloride at concentrations of 10, 25, and 50 μM were 93.5 ± 6.68%, 85.03 ± 4.99%, and 78.97 ± 7.04% of Con, respectively (Figure 4B). These data indicate that palmatine chloride can inhibit the excessive production of hydrogen peroxide in activated macrophages in a dose-dependent manner. The production of ROS, such as hydrogen peroxide, can also be increased by unfolded protein response [29,30,31]. ROS are known to exacerbate ER stress and cause apoptosis, leading to neurodegenerative diseases, atherosclerosis, diabetes mellitus, and inflammation in severe cases [29,30,31]. Like RNS, ROS could also destroy infectious microorganisms. However, they can cause toxicity to cells and damage surrounding tissues. Results of this experiment suggest that PA might be able to relieve endotoxin-induced cellular oxidative stress, ER stress, and damage to surrounding tissues by inhibiting the excessive production of ROS in pyroptotic macrophages. Baicalein was included as a reference compound for comparison with PA.

### 2.5. Cytokine Production

**Figure 5:**
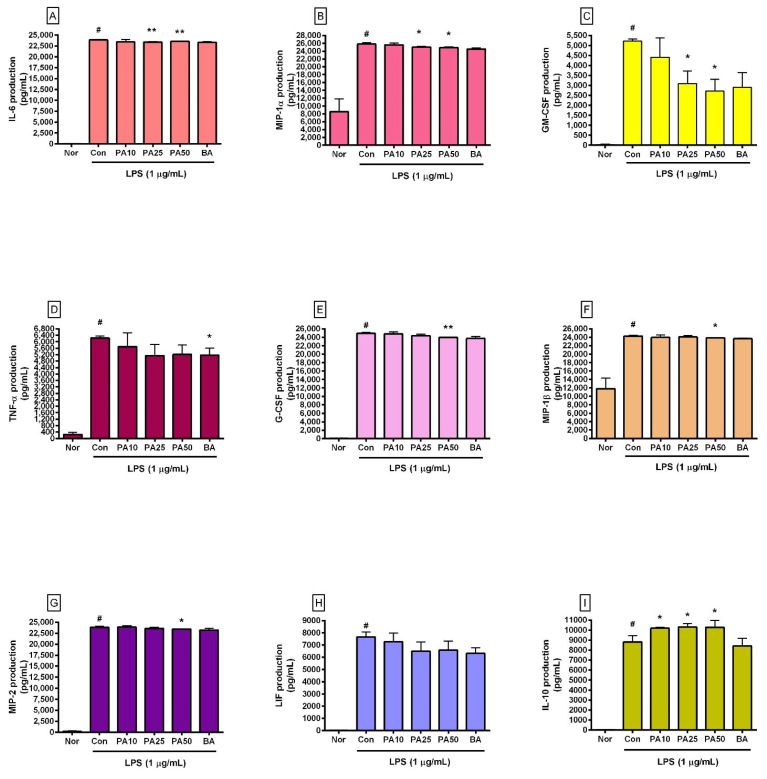
Production of IL-6 (**A**), MIP-1α (**B**), GM-CSF (**C**), TNF-α (**D**), G-CSF (**E**), MIP-1β (**F**), MIP-2 (**G**), LIF (**H**), and IL-10 (**I**) in lipopolysaccharide (LPS)-stimulated RAW 264.7 cells. Values are presented as mean ± standard deviation. Nor, the group treated with media alone; Con, the group treated with LPS alone. PA10 means 10 μM of palmatine chloride, PA25 means 25 μM of palmatine chloride, and PA50 means 50 μM of palmatine chloride. BA, baicalein (25 µM). ^#^, *p* < 0.05 vs. Nor; *, *p* < 0.05 vs. Con; **, *p* < 0.01 vs. Con.

Palmatine chloride reduced the production levels of various cytokines in RAW 264.7 stimulated by LPS. Specifically, palmatine chloride at concentrations of 25 and 50 μM significantly decreased production levels of IL-6, granulocyte macrophage colony-stimulating factor (GM-CSF; CSF2), and macrophage inflammatory proteins (MIP)-1α (CCL3). Palmatine chloride at a concentration of 50 μM significantly decreased the production of granulocyte colony-stimulating factor (G-CSF; CSF3), MIP-1β (CCL4), and MIP-2 (CXCL2). Additionally, palmatine chloride at concentrations of 10, 25, and 50 μM significantly increased the production of IL-10, a well-known anti-inflammatory cytokine (Figure 5, Table 1). In detail, in endotoxin-stimulated RAW 264.7 treated with palmatine chloride at concentrations of 10, 25, and 50 µM, production levels of IL-6 were 98 ± 2.28%, 97.92 ± 0.52%, and 98.47% ± 0.29% of Con, respectively; those of tumor necrosis factor (TNF)-α were 91.67 ± 13.71%, 82.6 ± 11.28%, and 83.86 ± 9.35% of Con, respectively; those of GM-CSF were 84.6 ± 18.52%, 59.28 ± 12.08%, and 51.89 ± 11.62% of Con, respectively; those of G-CSF were 99.43 ± 1.87%, 97.61 ± 1.7%, and 96.06 ± 0.14% of Con, respectively; those of MIP-1α were 99.27 ± 1.84%, 96.99 ± 0.82%, and 96.37 ± 0.82% of Con, respectively; those of MIP-1β were 98.7 ± 2.45%, 99.04 ± 1.27%, and 98.19 ± 0.27% of Con, respectively; those of MIP-2 were 100.23 ± 1.23%, 98.99 ± 0.93%, and 98.29 ± 0.19% of Con, respectively; those of LIF were 94.89 ± 9.2%, 84.87 ± 9.73%, and 86.13 ± 9.39% of Con, respectively; and those of IL-10 were 115.5 ± 1.12%, 116.97 ± 3.93%, and 116.54 ± 7.73% of Con, respectively. Contrary to the experimental results of measuring the production of NO and hydrogen peroxide, the decrease in cytokine production by PA10 (10 μM of PA) was not statistically significant. It seems that further research is needed on this. Baicalein was included as a reference compound for comparison with PA in the cytokine assay.

### 2.6. Transcriptions of Inflammatory Genes

**Figure 6:**
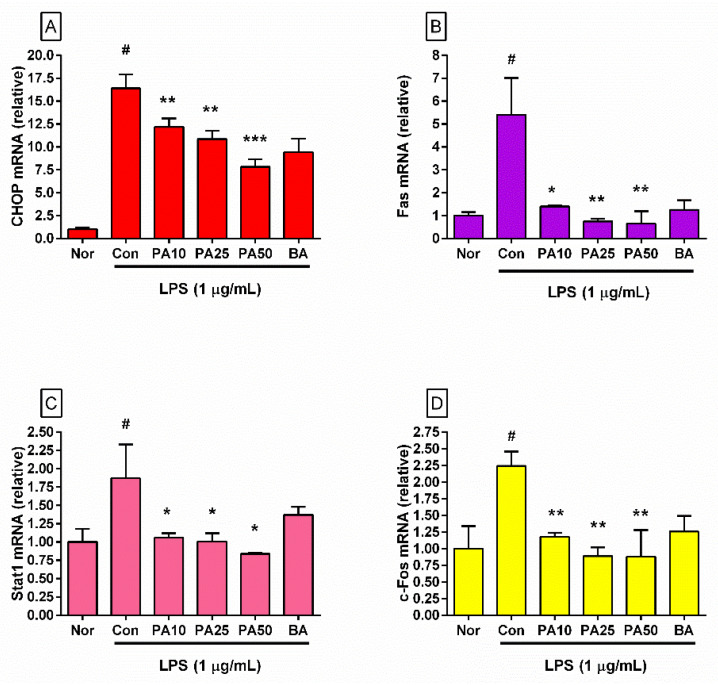
Effects of palmatine chloride on transcription levels of *Chop* (**A**), Fas (**B**), *Stat1* (**C**), and *c-Fos* (**D**) in lipopolysaccharide (LPS)-stimulated RAW 264.7 cells. Values are presented as mean ± standard deviation. Nor, the group treated with media alone; Con, the group treated with LPS alone. PA10 means 10 μM of palmatine chloride, PA25 means 25 μM of palmatine chloride, and PA50 means 50 μM of palmatine chloride. BA, baicalein (25 µM). ^#^, *p* < 0.05 vs. Nor; *, *p* < 0.05 vs. Con; **, *p* < 0.01 vs. Con; ***, *p* < 0.001 vs. Con.

Palmatine chloride inhibited excessive mRNA expression of *Stat1,* C*hop*, *Fas*, and *c-Fos* in LPS-activated RAW 264.7 (Figure 6, Table 1). In LPS-stimulated RAW 264.7 cells treated with PA at concentrations of 10, 25, and 50 µM, transcription levels of *Stat1* were 56.32 ± 3.13%, 53.67 ± 5.61%, and 44.98 ± 0.48% of Con, respectively; those of C*hop* were 74.46 ± 5.62%, 66.28 ± 5.66%, and 47.74 ± 5.02% of Con, respectively; those of *Fas* were 25.93 ± 0.73%, 13.9 ± 2.19%, and 12.03 ± 9.94% of Con, respectively; and those of *c-Fos* were 52.84 ± 2.75%, 39.99 ± 5.66%, and 39.23 ± 17.68% of Con, respectively. Interestingly, c-Fos is a well-known AP-1 transcription factor. AP-1 might be activated in response to various cell stimuli, including cytokines, oxidative stress, and infections, via p38 MAPK signaling, consequently triggering the CHOP pathway [36,37,38]. Additionally, AP-1-induced CHOP, an important ER stress transcription factor, can increase the release of calcium from ER stores of macrophages stimulated by infectious pathogens, resulting in apoptosis due to ER stress, which can increase the expression of Fas and activate STAT1 [12]. Our results suggest that palmatine chloride can relieve ER stress by suppressing the expression of inflammatory genes such as C*hop*, *stat1*, *Fas*, and c-*Fos* in RAW 264.7 cells stimulated by endotoxins via the CHOP pathway. Baicalein was included as a reference compound in the gene expression experiments.

### 2.7. Phosphorylation of p38 MAPK and Level of Fas

**Figure 7:**
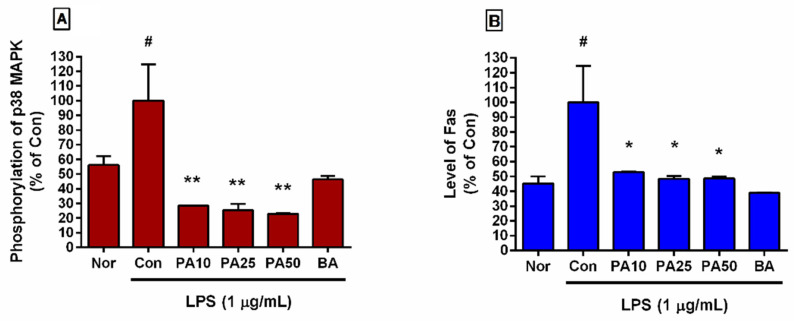
Effects of palmatine chloride on p38 MAPK phosphorylation (**A**) and level of Fas (**B**) in lipopolysaccharide (LPS)-stimulated RAW 264.7 cells. Values are presented as mean ± standard deviation. Nor, the group treated with media alone; Con, the group treated with LPS alone. PA10 means 10 μM of palmatine chloride, PA25 means 25 μM of palmatine chloride, and PA50 means 50 μM of palmatine chloride. BA, baicalein (25 µM). ^#^, *p* < 0.05 vs. Nor; *, *p* < 0.05 vs. Con; **, *p* < 0.01 vs. Con.

Phosphorylation levels of p38 MAPK in LPS-stimulated RAW 264.7 cells treated with palmatine chloride at concentrations of 10, 25, and 50 μM were 28.4 ± 0.13%, 25.28 ± 4.39%, and 22.77 ± 0.68% of Con, respectively (Figure 7A). Thus, PA could significantly inhibit the activation of p38 MAPK in a dose-dependent manner. Meanwhile, levels of Fas in endotoxin-stimulated RAW 264.7 cells treated with palmatine chloride at concentrations of 10, 25, and 50 μM were 52.88 ± 0.25%, 48.36 ± 1.92%, and 48.55 ± 1.45% of Con, respectively (Figure 7B). These results mean that PA could inhibit the programmed cell death caused by LPS in a concentration-dependent manner. Baicalein was included as a reference compound in the p38 MAPK and Fas analyses.

Macrophages stimulated by endotoxins undergo lytic programmed cell death as ER stress increases and CHOP activation proceeds, including excessive production of inflammatory mediators such as cytokines and NO that occurs through p38 MAPK activation accompanied by increased Fas expression [18,19,20,21]. This experimental result indicates that palmatine chloride can inhibit p38 MAPK activation of endotoxin-stimulated macrophages and alleviate ER stress in activated macrophages, thus suppressing the overproduction of pro-inflammatory mediators.

## 3. Discussion

Although many antibiotics have been developed, bacterial infections are still threatening human health. Therefore, many studies are being conducted on the treatment of bacterial infectious diseases using natural products [3,4]. Additionally, many studies have shown that natural products can relieve excessive inflammation caused by infection by decreasing the production of inflammatory mediators [5,6,7]. Since various inflammatory mediators are overproduced in the process of macrophage activation caused by infection pathogens, natural products that can suppress the overproduction of inflammatory mediators are thought to be able to regulate macrophage activation caused by infection. In a previous study, our research group reported that berberine can modulate macrophage activation caused by polyinosinic-polycytidylic acid (a double-stranded RNA) and inhibit the excessive production of NO and cytokines [39]. Berberine, a benzylisoquinoline alkaloid, is one of the main components found in *Coptis chinensis* and *Phellodendron amurense*. Interestingly, palmatine (i.e., a protoberberine alkaloid) with a structure similar to berberine has also been found in *Coptis chinensis* and *Phellodendron amurense*. Traditionally, both *Coptis chinensis* [47] and *Phellodendron amurense* [48] have been used to treat inflammatory diseases. Thus, studies on protoberberine alkaloids, such as PA, on macrophage activation, an important process for inflammatory pathophysiological phenomena, are meaningful. Interestingly, various studies have reported pharmacological efficacies of palmatine [40,41,42,43,44,45,46]. In 2019, Long et al. reported that palmatine had various pharmacological effects, such as neuroprotection, anti-bacterial, anti-inflammation, and anti-cancer effects [40]. In 2020, Ekeuku et al. reported the protective effects of palmatine against metabolic syndrome associated with cardiovascular diseases and osteoarthritis due to the antioxidative activities of palmatine [41]. Wang et al. in 2017 reported that palmatine could protect gastric mucosa and inhibit gastric ulcers induced by acetic acid in rats [44]. In 2018, Zhang et al. suggested that palmatine could ameliorate mouse colitis induced by dextran sulfate sodium by regulating gut microbiota and suppressing tryptophan catabolism [45]. Ma et al. in 2016 reported that palmatine could reduce dysplastic change in gut tumorigenesis in ApcMin/+ mice and inflammatory cytokines such as IL-8, CSF2 (GM-CSF), and CSF3 (G-CSF) in gut inflammation [46]. In the current study, results showed that PA treatment at concentrations of 25 and 50 μM for 24 h significantly decreased excessive production of IL-6, CSF2 (GM-CSF), and CCL3 (MIP-1α) in RAW 264.7 cells stimulated by LPS endotoxins. Additionally, PA at a concentration of 50 μM significantly inhibited the production of CSF3 (G-CSF), CXCL2 (MIP-2), and CCL4 (MIP-1β). Although TNF-α levels tended to decrease, the difference was not statistically significant. This cytokine production regulation of PA means that PA can modulate macrophage activation caused by the stimulation of endotoxins, thereby alleviating excessive inflammatory response in macrophages stimulated by endotoxins.

In a previous study, our research group demonstrated that baicalin could inhibit the inflammatory response of LPS-stimulated RAW 264.7 macrophages through the calcium–CHOP pathway associated with ER stress [7]. Previous studies have also identified ER stress and calcium dysregulation as key mechanisms involved in endotoxin-induced macrophage activation [7,10,11,12,13,14,15,16,17]. Consistent with this concept, PA significantly reduced cytosolic calcium release and the expression of ER stress-associated genes in LPS-stimulated macrophages, suggesting a regulatory role of PA in the calcium–CHOP signaling pathway. Furthermore, PA significantly inhibited NO production, cytosolic calcium release, and the transcription of Chop, Fas, and Stat1. These findings suggest that PA may attenuate macrophage activation induced by endotoxins by suppressing ER stress-associated calcium signaling and downstream CHOP-mediated inflammatory responses. In addition to its effects on ER stress-associated signaling, PA significantly inhibited NO production in LPS-stimulated macrophages. Excessive NO production is a characteristic feature of macrophage activation and has been implicated in ER stress amplification through disruption of calcium homeostasis and CHOP activation [22,23,24,25,26,27,28]. Therefore, the inhibitory effect of PA on NO production may contribute to its ability to attenuate ER stress-associated inflammatory responses. Together with its effects on cytosolic calcium release and CHOP-related gene expression, these findings further support a regulatory role of PA in the calcium–CHOP signaling pathway. Consistent with previous studies linking ER stress to calcium dysregulation, oxidative stress, and pro-apoptotic signaling [10], PA significantly reduced cytosolic calcium release, hydrogen peroxide production, Stat1 transcription, and Fas expression in LPS-stimulated macrophages. These findings suggest that PA may attenuate ER stress-associated inflammatory responses by modulating multiple downstream events related to the calcium–CHOP signaling cascade.

Of course, macrophages stimulated by infectious pathogens such as endotoxins will go through apoptosis, a lytic programmed cell death process involving inflammatory reactions such as generating and releasing large amounts of cytokines like IL-6 and TNF-α, which is different from general apoptosis [33,34,35]. In the present study, PA at concentrations of 10, 25, and 50 μM significantly inhibited NO production and cytosolic calcium release in LPS-stimulated RAW 264.7 as well as excessive transcription of *Chop*, *Fas*, and *Stat1*. These data suggest that PA might modulate macrophage activation induced by endotoxins through ER-stress-related calcium signaling and the CHOP pathway. The inclusion of THP-1 cells in the initial experiments provided preliminary support for the biological activity of PA in a human-derived immune cell line, whereas the detailed mechanistic analyses were performed in RAW 264.7 macrophages because of their extensive use in studies of ER stress and inflammatory signaling pathways. The production of ROS is also increased by an unfolded protein response, exacerbating ER stress, causing apoptosis, and leading to neurodegenerative diseases, atherosclerosis, diabetes mellitus, and inflammation in severe cases [29,30,31]. Our data presented that PA significantly reduced the excessive production of hydrogen peroxide in LPS-induced RAW 264.7. It is well known that ROS can stimulate p38 MAPK signaling [43]. Furthermore, Li et al. [18] have reported that LPS-induced macrophage activation involves massive production of pro-inflammatory cytokines through p38 MAPK signaling. Consistent with previous reports implicating p38 MAPK in ER stress-associated CHOP activation [19], the present study demonstrated that PA markedly suppressed p38 MAPK phosphorylation and reduced Chop transcription in LPS-stimulated macrophages. These findings suggest that inhibition of the p38 MAPK–CHOP axis may contribute to the anti-inflammatory activity of PA and may underlie its ability to attenuate cytokine production and ER stress-associated responses. Endotoxin-induced lung inflammation is exacerbated by the inflammatory response of endotoxin-stimulated macrophages caused by ER stress and CHOP activated by p38 MAPK [20]. Our data showed that PA inhibited p38 MAPK activation in LPS-stimulated RAW 264.7 cells, supporting the possibility that suppression of p38 MAPK-dependent inflammatory signaling contributes to its anti-inflammatory effects. ROS and cytokines in LPS-stimulated macrophages are well known to activate AP-1 via p38 MAPK signaling, consequently triggering the CHOP pathway [36,37,38]. The present study also demonstrated that PA significantly suppressed c-Fos transcription together with reductions in Chop, Stat1, and Fas expression. Given the reported role of AP-1/c-Fos in promoting CHOP-dependent ER stress signaling [12], these results suggest that inhibition of c-Fos-mediated transcriptional responses may contribute to the regulatory effects of PA on the ER stress-associated inflammatory cascade.

**Figure 8:**
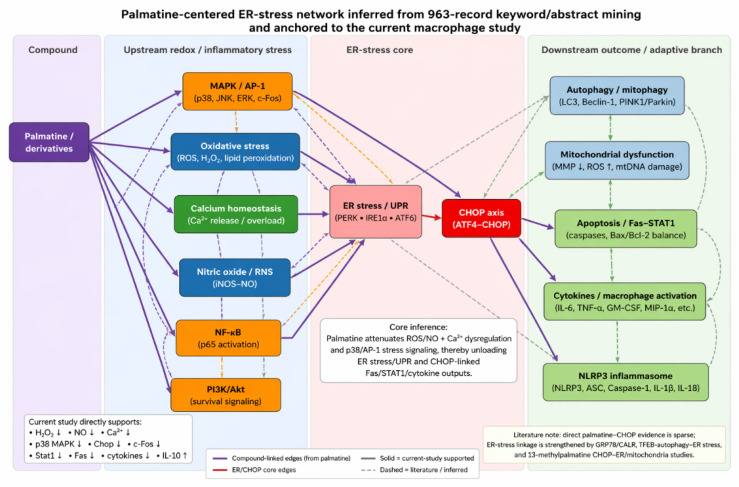
Proposed mechanism of palmatine chloride (PA) in ER stress-associated inflammatory responses. PA suppresses calcium–CHOP signaling and related inflammatory pathways, resulting in reduced macrophage activation and pro-inflammatory mediator production.

PA has not been widely characterized as a direct regulator of CHOP-mediated ER stress in previous studies. However, accumulating evidence from the palmatine literature indicates that its pharmacological actions converge on multiple upstream and downstream components of the ER stress network, including oxidative stress, calcium homeostasis, MAPK signaling, apoptosis, and autophagy (Figure 8).

These findings suggest that PA acts as a multi-target regulator of ER stress-associated inflammatory responses, an interpretation further supported by the network pharmacology analysis. Notably, the present study provides the first experimental evidence linking PA to the calcium–CHOP axis in macrophages, thereby bridging a critical gap between previously reported antioxidant and anti-inflammatory activities and ER stress-mediated cell death mechanisms, as summarized in Figure 8.

Despite these findings, the present study was limited to in vitro macrophage models. The effects of PA on STAT1/CAMKII signaling and extracellular calcium influx were not evaluated. It would also be meaningful to investigate the effects of PA on extracellular calcium influx and the activation of stromal interaction molecules in future studies. In the present study, baicalein and indomethacin were included as reference compounds to provide comparative benchmarks for the anti-inflammatory activity of palmatine chloride. Indomethacin, a well-established non-steroidal anti-inflammatory drug, effectively suppressed NO production, while baicalein, a flavonoid previously reported to inhibit macrophage activation through ER stress-related pathways, reduced inflammatory mediator production and ER stress-associated responses. Although direct comparisons among the compounds were beyond the scope of this study, the responses observed with baicalein and indomethacin served as useful benchmarks for interpreting the biological activity of palmatine chloride. Taken together, these findings support the potential role of PA as a modulator of ER stress-associated inflammatory responses and provide a basis for future in vivo and mechanistic studies.

## 4. Materials and Methods

### 4.1. Materials

Dulbecco’s modified Eagle medium, phosphate-buffered saline, LPS, baicalein, and indomethacin were purchased from Millipore (Billerica, MA, USA). Although PA and palmatine are structurally similar, PA is more water-soluble than palmatine due to the presence of the positively charged quaternary ammonium ion, so PA was purchased from Millipore and used for experiments in this study. The experimental concentration (10~50 μM) of PA was set by referring to previous studies [49,50] using palmatine (10~100 mg/L). Baicalein was selected as a comparative substance because it had already been reported to alleviate the inflammation of macrophages induced by LPS [51].

### 4.2. Cell Viability

Cells from the THP-1 human monocyte cell line (passage number 2) and RAW 264.7 mouse macrophage cell line (passage number 2) were obtained from the Korea Cell Line Bank (Seoul, Republic of Korea). THP-1 human monocytic cells were included to provide preliminary evidence of the biological activity of palmatine chloride in a human-derived immune cell model. Therefore, THP-1 cells were used for the initial assessment of cytotoxicity and NO production. Subsequent mechanistic experiments were conducted using RAW 264.7 macrophages, which are widely used for studies of macrophage activation, ER stress-associated signaling, and inflammatory responses. Cells were incubated with PA for 24 h in 96-well plates (1 × 10^4^ cells/well) to verify the toxicity of PA. After 24 h incubation with PA, cell viability was determined with a modified MTT assay [5,6,7]. The optical density (OD) was determined at 540 nm with a Model 680 microplate reader (Bio-Rad, Hercules, CA, USA).

### 4.3. NO Production, Cytosolic Calcium Level, and Hydrogen Peroxide Production

NO production was measured using the Griess assay. Cells (1 × 10^4^ cells/well) were treated with LPS and/or PA for 24 h. After incubation, culture supernatants were collected, and 100 µL of each supernatant was mixed with 100 µL of Griess reagent in a 96-well plate. After 15 min of incubation at room temperature, the OD of each well was determined at 540 nm. Intracellular calcium levels were measured using a Fluo-4 calcium assay. After the experimental treatment, cells (1 × 10^5^ cells/well) were incubated with 100 µL of Fluo-4 dye-loading solution for 30 min at 37 °C. After incubation, the fluorescence intensity of each well was determined using a spectrofluorometer (Dynex, West Sussex, UK) with excitation and emission filters of 485 nm and 535 nm, respectively. The production of hydrogen peroxide in cells was measured with the dihydrorhodamine (DHR) 123 Assay. Briefly, an aliquot of DHR was added to each well of a 96-well plate and pre-incubated at 37 °C for 30 min. The medium was then removed, and cells were incubated with LPS and/or PA for 24 h or 48 h at 37 °C. After incubation, the fluorescence intensities of wells were measured by a spectrofluorometer (Dynex) with an excitation filter at 485 nm and an emission filter at 535 nm. In all experiments, PA and LPS were added simultaneously without a pretreatment period.

### 4.4. Cytokine Production

Cytokines produced by RAW 264.7 after 24 h of treatment were evaluated with MILLIPLEX MAP Mouse Cytokine/Chemokine Magnetic Bead Panel kits (Millipore). RAW 264.7 were seeded into 96-well plates (1 × 10^4^ cells/well) and treated with LPS and/or PA. After 24 h of treatment, levels of the following cytokines in each well were analyzed. Briefly, the following procedures were performed. After pre-wetting a 96-well plate with Wash Buffer, the Wash Buffer was removed from each well using a Handheld Magnetic Separation Block (HMSB) (Milipore). Next, 25 µL of cell culture supernatant from each well was incubated with antibody-conjugated beads on a plate shaker for 2 h at room temperature. After incubation, well contents were gently removed with an HMSB, and the 96-well plate was washed twice. Then, 25 µL of Detection Antibodies was added to each well and incubated at room temperature with agitation on a plate shaker for 1 h. Subsequently, 25 µL Streptavidin–Phycoerythrin was added to each well containing Detection Antibodies and incubated for 30 min with agitation on a plate shaker at room temperature. After incubation, the well contents were gently removed and washed twice with HMSB. Then, 150 µL of Sheath Fluid was added to each well. Beads bound to each cytokine were analyzed with a Bio-Plex 200 instrument (Bio-Rad).

### 4.5. Transcripts of Inflammatory Genes

The mRNA levels of target genes such as *Chop*, *Stat1*, *Fas*, *c-Fos*, and *β-Actin* were quantified with real-time PCR using a Bio-Rad CFX 96 (Bio-Rad).

#### 4.5.1. Isolation of RNA

RAW 264.7 mouse macrophages were incubated with LPS and/or PA for 18 h in 6-well plates (1 × 10^6^ cells/well). After 18 h of incubation, total RNA for each well of cells was isolated using a NucleoSpin RNA kit (Macherey-Nagel, Duren, Germany). Briefly, 350 μL Lysis Buffer RA1 and 3.5 μL β-mercaptoethanol were added to the cell pellet and vigorously vortexed to lyse cells. Lysate was cleared by filtration using a NucleoSpin^®^ Filter (Macherey-Nagel). After 350 μL ethanol (70%) was added, cells were mixed by vortexing. Cell lysate was loaded into a NucleoSpin^®^ RNA Column (Macherey-Nagel). After 350 μL Membrane Desalting Buffer was added, the column was centrifuged. Then, 95 μL DNase reaction mixture was applied directly to the center of the silica membrane of the column, followed by incubation at room temperature for 15 min. Columns were washed with Wash Buffer RA2 and Wash Buffer RA3 (Macherey-Nagel). After the silica membrane was dried, RNA was eluted with 60 μL RNase-free water and centrifuged.

#### 4.5.2. Determination of RNA Concentration

RNA concentration was measured using an Experion RNA StdSens Analysis kit (Bio-Rad) with an Experion Automatic Electrophoresis System (Bio-Rad). First, electrodes were cleaned using a cleaning chip filled with 900 μL DEPC-treated water. After a gel-stain solution was prepared, 9 μL of the solution was added to the labeled wells, and the chip was primed. Samples and RNA ladder were loaded into the chip, which was then vortexed using an Experion vortex station for 1 min. Lastly, the chip was loaded into the electrophoresis platform, and an RNA StdSens Analysis program was run.

#### 4.5.3. cDNA Synthesis

cDNAs were synthesized using RNA samples and an iScript cDNA Synthesis kit (Bio-Rad). Briefly, 20 μL complete reaction mixes were prepared using 5× iScript Reaction Mix (4 μL), iScript Reverse Transcriptase (1 μL), Nuclease-free water (variable), and RNA template (variable, 1 μg total RNA). The reaction mix (20 μL) was incubated in a thermal cycler (C1000 Thermal Cycler, Bio-Rad), according to the manufacturer’s protocol (priming at 25 °C for 5 min, reverse transcription at 46 °C for 20 min, and RT inactivation at 95 °C for 1 min).

#### 4.5.4. Quantitative Real-Time PCR Analysis

Gene expression was measured using a quantitative polymerase chain reaction with an iQ SYBR Green Supermix (Bio-Rad) and a CFX96 Real-Time PCR Detection System (Bio-Rad). Briefly, a master mix was prepared for all reactions by adding iQ SYBR Green Supermix and forward/reverse primers for each target gene. This master mix was thoroughly mixed to ensure homogeneity. Then, 7 μL was dispensed into each well of a qPCR plate. Next, 3 μL of cDNA was added to each well. Any air bubbles in the vessel bottom were removed. The PCR plate was loaded into the real-time PCR instrument. PCR was performed using the following cycling conditions: denaturation of DNA at 95 °C for 3 min, followed by 40 cycles of 95 °C for 10 s and 55 °C for 30 s. Relative mRNA expression levels were normalized against *β-actin* as an internal control and calculated with the 2^−Δ∆Ct^ (Ct: cycle threshold) method. Table 2 lists the primers used in this study.

### 4.6. Flow Cytometric Analysis for Levels of Phosphorylated p38 MAPK and Fas

RAW 264.7 was incubated with LPS and/or PA for 18 h in 6-well plates (3 × 10^5^ cells/well). Phosphorylated p38 MAPK and Fas levels in RAW 264.7 were then evaluated via flow cytometry using an Attune NxT flow cytometer (Thermo Fisher Scientific, Waltham, MA, USA). Briefly, after 18 h of treatment, cells were stained with Fixable Viability Dye eFluor 520 (eBioscience 65-0867-18, San Diego, CA, USA), phospho-p38 MAPK (T180/Y182) Monoclonal Antibody (eBioscience 17-9078-42), CD95 (APO-1/Fas) Monoclonal Antibody (eBioscience 12-0951-83), mouse IgG1 kappa isotype control (eBioscience 12-4714-81), and mouse IgG2b kappa isotype control (eBioscience 12-4732-81) according to the manufacturer’s protocol. Fixable Viability Dye eFluor 520 was used to irreversibly label dead cells prior to cryopreservation, fixation, and/or permeabilization procedures. Cells were fixed with a Fix Buffer (Thermo Fisher Scientific), permeabilized with a Perm Buffer (Thermo Fisher Scientific), and stained with fluorescent-labeled antibodies. Stained cells were analyzed with an Attune NxT flow cytometer. A serial gating strategy used forward scatter versus side scatter plots, forward scatter versus viability stain plots, and target antibody expression plots. Unstained cells were used as negative controls for gating. Data were obtained from the mean fluorescent intensities of samples. Details regarding startup, proper calibration, and operation of the Attune NxT flow cytometer can be found in the Attune User Guide (https://assets.thermofisher.com/TFS-Assets/LSG/manuals/100024235_AttuneNxT_HW_UG.pdf, accessed on 4 May 2026). PA and LPS were added simultaneously to the cells, and no pretreatment period was applied.

### 4.7. Statistical Analyses

Data are representatives of at least three independent experiments. Values are expressed as means ± standard deviation (SD). All data were subjected to a one-way analysis of variance (ANOVA) followed by Tukey’s multiple comparison test using GraphPad Prism (version 4; GraphPad Software, San Diego, CA, USA).

### 4.8. Network Pharmacology Analysis

To explore the potential mechanisms of PA beyond the experimental findings, a network pharmacology-informed analysis was conducted based on literature mining. A total of 963 palmatine-related articles were collected and curated from published literature sources, and the full list is provided in Supplementary Table S1. Keywords and abstracts were analyzed to identify frequently co-occurring biological processes, signaling pathways, and molecular targets associated with palmatine. Target-related terms were manually standardized and categorized into functional groups, including oxidative stress, inflammatory signaling, apoptosis, autophagy, and endoplasmic reticulum (ER) stress-related pathways. Based on these data, a compound–target–pathway interaction network was constructed. Key nodes were selected according to their frequency of occurrence and biological relevance to macrophage activation and ER stress. The network was visualized using Cytoscape software (version 3.10.4), and node–edge relationships were defined to represent interactions among palmatine, molecular targets, and signaling pathways. In addition, a mock enrichment analysis was performed to identify representative Gene Ontology terms and Kyoto Encyclopedia of Genes and Genomes pathways associated with the extracted targets. This analysis was conducted as a literature-informed approximation to support mechanistic interpretation rather than a database-driven enrichment test. The dataset was used for qualitative network inference rather than quantitative meta-analysis. In the preparation of this section, a generative artificial intelligence tool (ChatGPT 5.0, OpenAI, San Francisco, CA, USA) was used to assist in organizing literature-derived information and drafting descriptive text related to network pharmacology. The identification of key pathways, interpretation of biological mechanisms, and all scientific conclusions were independently performed and critically evaluated by the authors.

## 5. Conclusions

PA exerts anti-inflammatory effects on endotoxin-stimulated RAW 264.7 cells via the calcium–CHOP pathway, consequently reducing the endotoxin-induced production of pro-inflammatory mediators (e.g., NO and cytokines) and alleviating ER stress-related pyroptotic cascade, highlighting its potential as a multi-target therapeutic agent for ER stress-associated inflammatory diseases.

## Figures and Tables

**Table 1:** Effects of palmatine chloride on lipopolysaccharide-activated RAW 264.7 cells.

Inflammatory Factor	Normal (Media Only)			Control (LPS Alone)			Concentration (μM) of Palmatine Chloride with Lipopolysaccharide (1 µg/mL)								
							10			25			50		
IL-6 (pg/mL)	47.00	±	7.39	23,915.00	±	134.41	23,437.50	±	544.91	23,416.50	±	125.52 **	23,548.13	±	68.49 **
G-CSF (pg/mL)	79.67	±	23.16	24,929.00	±	280.38	24,787.83	±	467.42	24,332.00	±	422.63	23,947.83	±	34.40 **
IL-10 (pg/mL)	20.19	±	1.41	8813.88	±	614.64	10,180.00	±	98.46 *	10,309.83	±	346.82 *	10,271.67	±	681.40 *
MIP-1α (pg/mL)	8570.67	±	3279.51	25,817.33	±	378.72	25,629.17	±	475.58	25,039.67	±	211.60 *	24,879.50	±	210.72 *
MIP-2 (pg/mL)	204.17	±	119.47	23,859.63	±	247.86	23,915.67	±	293.74	23,618.67	±	223.01	23,451.50	±	44.92 *
TNF-α (pg/mL)	248.38	±	126.82	6222.83	±	136.28	5704.50	±	852.86	5140.00	±	701.95	5218.50	±	581.55
GM-CSF (pg/mL)	36.25	±	7.76	5215.00	±	97.44	4411.67	±	965.94	3091.33	±	629.91 *	2706.00	±	606.23 *
LIF (pg/mL)	29.00	±	3.91	7657.67	±	422.35	7266.33	±	704.40	6499.00	±	745.09	6595.17	±	719.13
MIP-1β (pg/mL)	11,810.17	±	2533.91	24,300.25	±	170.14	23,984.38	±	596.27	24067.75	±	308.82	23,861.00	±	66.05 *
*Chop* mRNA (fold change)	1.00	±	0.23	16.39	±	1.53	12.20	±	0.92 **	10.86	±	0.93 **	7.82	±	0.82 ***
*Fas* mRNA (fold change)	1.00	±	0.16	5.41	±	1.61	1.40	±	0.04 *	0.75	±	0.12 **	0.65	±	0.54 **
*Stat1* mRNA (fold change)	1.00	±	0.18	1.87	±	0.46	1.06	±	0.06 *	1.01	±	0.11 *	0.84	±	0.01 *
*c-Fos* mRNA (fold change)	1.00	±	0.34	2.24	±	0.22	1.18	±	0.06 **	0.89	±	0.13 **	0.88	±	0.40 **

Values are presented as mean ± standard deviation (*n* = 4). *, *p* < 0.05 vs. Con; **, *p* < 0.01 vs. Con; ***, *p* < 0.001 vs. Con.

**Table 2:** Primers used in quantitative real-time PCR.

Name ^1^ (GenBank Accession Number)	Forward Primer (5′–3′)	Reverse Primer (5′–3′)
*Chop* *(NM_007837)*	CCACCACACCTGAAAGCAG	TCCTCATACCAGGCTTCCA
*Fas* *(NM_009283.4)*	CGCTGTTTTCCCTTGCTG	CCTTGAGTATGAACTCTTAACTGTGAG
*Stat1* *(NM_007987)*	TGAGATGTCCCGGATAGTGG	CGCCAGAGAGAAATTCGTGT
*c-Fos* *(NM_010234)*	AGAGCGGGAATGGTGAAGA	TCTTCCTCTTCAGGAGATAGCTG
*β-Actin* *(NM_007393.3)*	CTAAGGCCAACCGTGAAAAG	ACCAGAGGCATACAGGGACA

^1^ Primer names: C/EBP homologous protein (*Chop*), First apoptosis signal receptor (*Fas*), Signal Transducer and Activator of Transcription 1 (*Stat1*), *c-Fos*, and *β-Actin*.

## Data Availability

The original contributions presented in this study are included in the article/Supplementary Materials. Further inquiries can be directed to the corresponding author(s).

## Supplementary Materials

The following supporting information can be downloaded at: https://www.mdpi.com/article/10.3390/ijms27135704/s1.
