# Supplementary material for: Anti-Inflammatory Effect of Palmatine Chloride on Lipopolysaccharide-Stimulated RAW 264.7 Mouse Macrophages via Calcium-CHOP Pathway
Source: Int J Mol Sci. 2026 Jun 24;27(13):5704. doi: 10.3390/ijms27135704 (PMC13362026; doi:10.3390/ijms27135704)
Supplement: Supplementary file 1 [file ijms-27-05704-s001.zip › Supplementary Table S1.pdf]

No
1
2
3
4
5
6
7
8
9
10
11
12
13
14
15
16
17
18
19
20
21
22
23
24
25
26
27
28
29
30
31
32
33
34
35
36
37
38
39
40
41
42

43
44
45
46
47
48
49
50
51
52
53
54
55
56
57
58
59
60
61
62
63
64
65
66
67
68
69
70
71
72
73
74
75
76
77
78
79
80
81
82
83
84
85

86
87
88
89
90
91
92
93
94
95
96
97
98
99
100
101
102
103
104
105
106
107
108
109
110
111
112
113
114
115
116
117
118
119
120
121
122
123
124
125
126
127
128

129
130
131
132
133
134
135
136
137
138
139
140
141
142
143
144
145
146
147
148
149
150
151
152
153
154
155
156
157
158
159
160
161
162
163
164
165
166
167
168
169
170
171

172
173
174
175
176
177
178
179
180
181
182
183
184
185
186
187
188
189
190
191
192
193
194
195
196
197
198
199
200
201
202
203
204
205
206
207
208
209
210
211
212
213
214

215
216
217
218
219
220
221
222
223
224
225
226
227
228
229
230
231
232
233
234
235
236
237
238
239
240
241
242
243
244
245
246
247
248
249
250
251
252
253
254
255
256
257

258
259
260
261
262
263
264
265
266
267
268
269
270
271
272
273
274
275
276
277
278
279
280
281
282
283
284
285
286
287
288
289
290
291
292
293
294
295
296
297
298
299
300

301
302
303
304
305
306
307
308
309
310
311
312
313
314
315
316
317
318
319
320
321
322
323
324
325
326
327
328
329
330
331
332
333
334
335
336
337
338
339
340
341
342
343

344
345
346
347
348
349
350
351
352
353
354
355
356
357
358
359
360
361
362
363
364
365
366
367
368
369
370
371
372
373
374
375
376
377
378
379
380
381
382
383
384
385
386

387
388
389
390
391
392
393
394
395
396
397
398
399
400
401
402
403
404
405
406
407
408
409
410
411
412
413
414
415
416
417
418
419
420
421
422
423
424
425
426
427
428
429

430
431
432
433
434
435
436
437
438
439
440
441
442
443
444
445
446
447
448
449
450
451
452
453
454
455
456
457
458
459
460
461
462
463
464
465
466
467
468
469
470
471
472

473
474
475
476
477
478
479
480
481
482
483
484
485
486
487
488
489
490
491
492
493
494
495
496
497
498
499
500
501
502
503
504
505
506
507
508
509
510
511
512
513
514
515

516
517
518
519
520
521
522
523
524
525
526
527
528
529
530
531
532
533
534
535
536
537
538
539
540
541
542
543
544
545
546
547
548
549
550
551
552
553
554
555
556
557
558

559
560
561
562
563
564
565
566
567
568
569
570
571
572
573
574
575
576
577
578
579
580
581
582
583
584
585
586
587
588
589
590
591
592
593
594
595
596
597
598
599
600
601

602
603
604
605
606
607
608
609
610
611
612
613
614
615
616
617
618
619
620
621
622
623
624
625
626
627
628
629
630
631
632
633
634
635
636
637
638
639
640
641
642
643
644

645
646
647
648
649
650
651
652
653
654
655
656
657
658
659
660
661
662
663
664
665
666
667
668
669
670
671
672
673
674
675
676
677
678
679
680
681
682
683
684
685
686
687

688
689
690
691
692
693
694
695
696
697
698
699
700
701
702
703
704
705
706
707
708
709
710
711
712
713
714
715
716
717
718
719
720
721
722
723
724
725
726
727
728
729
730

731
732
733
734
735
736
737
738
739
740
741
742
743
744
745
746
747
748
749
750
751
752
753
754
755
756
757
758
759
760
761
762
763
764
765
766
767
768
769
770
771
772
773

774
775
776
777
778
779
780
781
782
783
784
785
786
787
788
789
790
791
792
793
794
795
796
797
798
799
800
801
802
803
804
805
806
807
808
809
810
811
812
813
814
815
816

817
818
819
820
821
822
823
824
825
826
827
828
829
830
831
832
833
834
835
836
837
838
839
840
841
842
843
844
845
846
847
848
849
850
851
852
853
854
855
856
857
858
859

860
861
862
863
864
865
866
867
868
869
870
871
872
873
874
875
876
877
878
879
880
881
882
883
884
885
886
887
888
889
890
891
892
893
894
895
896
897
898
899
900
901
902

903
904
905
906
907
908
909
910
911
912
913
914
915
916
917
918
919
920
921
922
923
924
925
926
927
928
929
930
931
932
933
934
935
936
937
938
939
940
941
942
943
944
945

946
947
948
949
950
951
952
953
954
955
956
957
958
959
960
961
962
963

Journal
Lloydia
Lloydia
Lloydia
Lloydia
Journal of pharmaceutical sciences
Chemical & pharmaceutical bulletin
Zhongguo Zhong yao za zhi = Zhongguo zhongyao zazhi = China journal of Chinese materia me
Zhongguo Zhong yao za zhi = Zhongguo zhongyao zazhi = China journal of Chinese materia me
Journal of ethnopharmacology
Yao xue xue bao = Acta pharmaceutica Sinica
Gaoxiong yi xue ke xue za zhi = The Kaohsiung journal of medical sciences
Yao xue xue bao = Acta pharmaceutica Sinica
Journal of medicinal chemistry
Acta anatomica
European journal of biochemistry
Yao xue xue bao = Acta pharmaceutica Sinica
Yao xue xue bao = Acta pharmaceutica Sinica
Farmatsiia
Yao xue xue bao = Acta pharmaceutica Sinica
Pharmaceutisch weekblad
Journal of pharmaceutical sciences
Chemical & pharmaceutical bulletin
Yakugaku zasshi : Journal of the Pharmaceutical Society of Japan
Molekuliarnaia biologiia
Cancer letters
Japanese journal of pharmacology
Journal of chromatography. A
Zhongguo Zhong yao za zhi = Zhongguo zhongyao zazhi = China journal of Chinese materia me
Journal of chromatography. A
Planta medica
African journal of medicine and medical sciences
Phytochemistry
Planta medica
Biochemistry
Zhongguo Zhong yao za zhi = Zhongguo zhongyao zazhi = China journal of Chinese materia me
Biochemical pharmacology
Journal of chromatography. A
Planta medica
Life sciences
The Journal of antimicrobial chemotherapy
Archives of pharmacal research
Biological & pharmaceutical bulletin

Journal of agricultural and food chemistry
Analytical chemistry
Journal of chromatography. A
Analytical chemistry
Journal of pharmaceutical and biomedical analysis
Neurochemical research
Journal of agricultural and food chemistry
Journal of Tongji Medical University = Tong ji yi ke da xue xue bao
Pharmaceutica acta Helvetiae
Journal of economic entomology
Oral diseases
Phytochemistry
Analytical chemistry
Planta medica
Journal of natural products
Analytical chemistry
Zhong yao cai = Zhongyaocai = Journal of Chinese medicinal materials
Life sciences
IUBMB life
Yao xue xue bao = Acta pharmaceutica Sinica
Zhongguo Zhong yao za zhi = Zhongguo zhongyao zazhi = China journal of Chinese materia me
Zhongguo Zhong yao za zhi = Zhongguo zhongyao zazhi = China journal of Chinese materia me
European journal of biochemistry
Journal of agricultural and food chemistry
Life sciences
Bioscience, biotechnology, and biochemistry
Zhongguo Zhong yao za zhi = Zhongguo zhongyao zazhi = China journal of Chinese materia me
Zhongguo Zhong yao za zhi = Zhongguo zhongyao zazhi = China journal of Chinese materia me
World journal of gastroenterology
Rapid communications in mass spectrometry : RCM
Se pu = Chinese journal of chromatography
Planta medica
Journal of natural products
Zhongguo Zhong yao za zhi = Zhongguo zhongyao zazhi = China journal of Chinese materia me
Photochemistry and photobiology
Journal of AOAC International
Di 1 jun yi da xue xue bao = Academic journal of the first medical college of PLA
Phytotherapy research : PTR
Comptes rendus hebdomadaires des seances de l'Academie des sciences
Archiv der Pharmazie und Berichte der Deutschen Pharmazeutischen Gesellschaft
Yao xue xue bao = Acta pharmaceutica Sinica
The American journal of Chinese medicine
Journal of agricultural and food chemistry

Phytochemistry
The Journal of pharmacy and pharmacology
Zhongguo Zhong yao za zhi = Zhongguo zhongyao zazhi = China journal of Chinese materia me
Journal of chromatographic science
Journal of chromatography. A
Bioorganic & medicinal chemistry letters
Journal of pharmaceutical and biomedical analysis
Phytotherapy research : PTR
Journal of mass spectrometry : JMS
Journal of natural products
Archives of pharmacal research
Zhongguo Zhong yao za zhi = Zhongguo zhongyao zazhi = China journal of Chinese materia me
Rapid communications in mass spectrometry : RCM
Zhongguo yi xue ke xue yuan xue bao. Acta Academiae Medicinae Sinicae
Journal of pharmaceutical and biomedical analysis
Journal of separation science
Bioorganic & medicinal chemistry
The Journal of veterinary medical science
The Journal of pharmacy and pharmacology
Journal of the American Society for Mass Spectrometry
Journal of pharmaceutical and biomedical analysis
Sheng wu gong cheng xue bao = Chinese journal of biotechnology
Journal of chromatography. A
Zhongguo Zhong yao za zhi = Zhongguo zhongyao zazhi = China journal of Chinese materia me
Journal of pharmaceutical and biomedical analysis
Electrophoresis
Journal of pharmaceutical and biomedical analysis
Yao xue xue bao = Acta pharmaceutica Sinica
Zhongguo Zhong yao za zhi = Zhongguo zhongyao zazhi = China journal of Chinese materia me
Bioorganic & medicinal chemistry letters
Chemical research in toxicology
Journal of pharmaceutical and biomedical analysis
Phytomedicine : international journal of phytotherapy and phytopharmacology
The Journal of pharmacy and pharmacology
International journal of biological macromolecules
Chemical research in toxicology
Journal of separation science
Yao xue xue bao = Acta pharmaceutica Sinica
Archives of pharmacal research
Plant foods for human nutrition (Dordrecht, Netherlands)
Zhong yao cai = Zhongyaocai = Journal of Chinese medicinal materials
Journal of separation science
Journal of pharmaceutical and biomedical analysis

Bioorganic & medicinal chemistry letters
Biophysical chemistry
Yao xue xue bao = Acta pharmaceutica Sinica
Zhongguo Zhong yao za zhi = Zhongguo zhongyao zazhi = China journal of Chinese materia me
Planta medica
Planta medica
Chemistry & biodiversity
Analytical chemistry
Planta medica
Planta medica
Planta medica
Planta medica
Planta medica
Biochimica et biophysica acta
Journal of chromatography. A
Analytica chimica acta
Rapid communications in mass spectrometry : RCM
Journal of separation science
Journal of chromatography. B, Analytical technologies in the biomedical and life sciences
Photochemistry and photobiology
FEBS letters
Journal of biochemical and biophysical methods
Biopharmaceutics & drug disposition
Journal of mass spectrometry : JMS
Journal of natural products
Electrophoresis
Photochemistry and photobiology
British journal of pharmacology
Journal of chromatography. B, Analytical technologies in the biomedical and life sciences
Biomedical chromatography : BMC
Archives of biochemistry and biophysics
Analytica chimica acta
Chemistry & biodiversity
Electrophoresis
Phytochemical analysis : PCA
Proceedings of the National Academy of Sciences of the United States of America
Phytotherapy research : PTR
Yao xue xue bao = Acta pharmaceutica Sinica
Zhongguo Zhong yao za zhi = Zhongguo zhongyao zazhi = China journal of Chinese materia me
Journal of natural products
Magnetic resonance in chemistry : MRC
DNA and cell biology
Journal of chromatography. A

Electrophoresis
Archives of pharmacal research
Talanta
Journal of pharmaceutical and biomedical analysis
The journal of physical chemistry. B
Planta medica
Electrophoresis
Analytica chimica acta
Zhongguo Zhong yao za zhi = Zhongguo zhongyao zazhi = China journal of Chinese materia me
Zhong yao cai = Zhongyaocai = Journal of Chinese medicinal materials
Journal of pharmaceutical and biomedical analysis
Journal of hazardous materials
Journal of pharmaceutical and biomedical analysis
Journal of separation science
Archives of pharmacal research
The Journal of pharmacy and pharmacology
Journal of ethnopharmacology
Cell biochemistry and function
Journal of AOAC International
Food and chemical toxicology : an international journal published for the British Industrial Biologi
Organic & biomolecular chemistry
Phytochemical analysis : PCA
Journal of ethnopharmacology
Zhongguo Zhong yao za zhi = Zhongguo zhongyao zazhi = China journal of Chinese materia me
Biological & pharmaceutical bulletin
Natural product research
Zhongguo Zhong yao za zhi = Zhongguo zhongyao zazhi = China journal of Chinese materia me
Zhongguo Zhong yao za zhi = Zhongguo zhongyao zazhi = China journal of Chinese materia me
Chinese medicine
DNA and cell biology
Evidence-based complementary and alternative medicine : eCAM
Luminescence : the journal of biological and chemical luminescence
Zhong xi yi jie he xue bao = Journal of Chinese integrative medicine
The journal of physical chemistry. B
Chemistry & biodiversity
Food and chemical toxicology : an international journal published for the British Industrial Biologi
Immunopharmacology and immunotoxicology
Zhongguo Zhong yao za zhi = Zhongguo zhongyao zazhi = China journal of Chinese materia me
Rapid communications in mass spectrometry : RCM
Biomacromolecules
Mutation research
Analytica chimica acta
Medicinal research reviews

Planta medica
Journal of pharmaceutical and biomedical analysis
Phytomedicine : international journal of phytotherapy and phytopharmacology
Journal of ethnopharmacology
Natural product research
Luminescence : the journal of biological and chemical luminescence
Zhongguo Zhong yao za zhi = Zhongguo zhongyao zazhi = China journal of Chinese materia me
Zhongguo Zhong yao za zhi = Zhongguo zhongyao zazhi = China journal of Chinese materia me
Planta medica
Journal of Asian natural products research
Biological & pharmaceutical bulletin
Journal of pharmaceutical and biomedical analysis
Archives of virology
Journal of chromatography. B, Analytical technologies in the biomedical and life sciences
Journal of chromatography. B, Analytical technologies in the biomedical and life sciences
Journal of AOAC International
Journal of chromatography. B, Analytical technologies in the biomedical and life sciences
Archives of pharmacal research
Zhongguo Zhong yao za zhi = Zhongguo zhongyao zazhi = China journal of Chinese materia me
Se pu = Chinese journal of chromatography
Journal of separation science
Journal of nucleic acids
Journal of natural products
Journal of Alzheimer's disease : JAD
Biomedical chromatography : BMC
Biological & pharmaceutical bulletin
Zhongguo Zhong yao za zhi = Zhongguo zhongyao zazhi = China journal of Chinese materia me
Zhongguo Zhong yao za zhi = Zhongguo zhongyao zazhi = China journal of Chinese materia me
Se pu = Chinese journal of chromatography
Journal of separation science
The Journal of allergy and clinical immunology
Biomedical chromatography : BMC
Journal of separation science
Zhongguo Zhong yao za zhi = Zhongguo zhongyao zazhi = China journal of Chinese materia me
Photochemistry and photobiology
Journal of natural medicines
Journal of separation science
Biochimica et biophysica acta
Xenobiotica; the fate of foreign compounds in biological systems
Planta
Journal of natural products
Phytotherapy research : PTR
Zhongguo Zhong yao za zhi = Zhongguo zhongyao zazhi = China journal of Chinese materia me

Advances in dental research
Journal of ethnopharmacology
Talanta
Spectrochimica acta. Part A, Molecular and biomolecular spectroscopy
Talanta
Phytomedicine : international journal of phytotherapy and phytopharmacology
Pharmaceutical research
The Analyst
Planta medica
Journal of pharmaceutical and biomedical analysis
Fitoterapia
Pharmacognosy magazine
Toxicology international
Journal of pharmaceutical and biomedical analysis
Zhongguo Zhong yao za zhi = Zhongguo zhongyao zazhi = China journal of Chinese materia me
Zhong yao cai = Zhongyaocai = Journal of Chinese medicinal materials
Zhongguo Zhong yao za zhi = Zhongguo zhongyao zazhi = China journal of Chinese materia me
Zhongguo Zhong yao za zhi = Zhongguo zhongyao zazhi = China journal of Chinese materia me
Zhongguo Zhong yao za zhi = Zhongguo zhongyao zazhi = China journal of Chinese materia me
Journal of pharmaceutical and biomedical analysis
Molecular biology reports
Analytical chemistry
Zhongguo Zhong yao za zhi = Zhongguo zhongyao zazhi = China journal of Chinese materia me
Phytotherapy research : PTR
The journal of physical chemistry. B
Planta medica
Zhongguo Zhong yao za zhi = Zhongguo zhongyao zazhi = China journal of Chinese materia me
Journal of ethnopharmacology
Zhongguo Zhong yao za zhi = Zhongguo zhongyao zazhi = China journal of Chinese materia me
Zhongguo Zhong yao za zhi = Zhongguo zhongyao zazhi = China journal of Chinese materia me
Die Pharmazie
Zhong yao cai = Zhongyaocai = Journal of Chinese medicinal materials
Nucleic acid therapeutics
Journal of chromatography. B, Analytical technologies in the biomedical and life sciences
Journal of ethnopharmacology
Food and chemical toxicology : an international journal published for the British Industrial Biologi
Journal of pharmaceutical and biomedical analysis
Journal of mass spectrometry : JMS
Zhongguo Zhong yao za zhi = Zhongguo zhongyao zazhi = China journal of Chinese materia me
Analytica chimica acta
Zhongguo Zhong yao za zhi = Zhongguo zhongyao zazhi = China journal of Chinese materia me
Zhongguo Zhong yao za zhi = Zhongguo zhongyao zazhi = China journal of Chinese materia me
Journal of natural medicines

Archives of pharmacal research
Journal of chromatography. B, Analytical technologies in the biomedical and life sciences
Journal of chromatography. B, Analytical technologies in the biomedical and life sciences
Analytica chimica acta
Molecules (Basel, Switzerland)
Zhongguo Zhong yao za zhi = Zhongguo zhongyao zazhi = China journal of Chinese materia me
Mitochondrion
Bioorganic & medicinal chemistry
Molecular biology reports
Current pharmaceutical design
Phytomedicine : international journal of phytotherapy and phytopharmacology
Journal of ethnopharmacology
Molecular bioSystems
Biochemistry. Biokhimiia
Journal of ethnopharmacology
Journal of AOAC International
Advances in pharmacological sciences
Phytomedicine : international journal of phytotherapy and phytopharmacology
Talanta
Evidence-based complementary and alternative medicine : eCAM
Phytomedicine : international journal of phytotherapy and phytopharmacology
Journal of mass spectrometry : JMS
Zhongguo Zhong yao za zhi = Zhongguo zhongyao zazhi = China journal of Chinese materia me
Rapid communications in mass spectrometry : RCM
Journal of chromatography. A
International journal of molecular sciences
Journal of chromatographic science
Journal of Asian natural products research
BMC complementary and alternative medicine
Se pu = Chinese journal of chromatography
Yao xue xue bao = Acta pharmaceutica Sinica
Journal of ethnopharmacology
Phytomedicine : international journal of phytotherapy and phytopharmacology
Biomedical chromatography : BMC
Evidence-based complementary and alternative medicine : eCAM
Zhongguo Zhong yao za zhi = Zhongguo zhongyao zazhi = China journal of Chinese materia me
Luminescence : the journal of biological and chemical luminescence
Zhongguo Zhong yao za zhi = Zhongguo zhongyao zazhi = China journal of Chinese materia me
Toxicology letters
Drug research
Evidence-based complementary and alternative medicine : eCAM
Applied spectroscopy
Toxicology and applied pharmacology

Analytica chimica acta
Food chemistry
Pakistan journal of pharmaceutical sciences
Analytical and bioanalytical chemistry
Integrative cancer therapies
Se pu = Chinese journal of chromatography
Planta medica
The journal of physical chemistry. B
Journal of alternative and complementary medicine (New York, N.Y.)
The Journal of pharmacy and pharmacology
Plant cell reports
Iranian journal of pharmaceutical research : IJPR
Plant cell reports
Journal of ethnopharmacology
Journal of chromatography. A
Journal of chromatography. B, Analytical technologies in the biomedical and life sciences
Spectrochimica acta. Part A, Molecular and biomolecular spectroscopy
Journal of pharmaceutical and biomedical analysis
Integrative cancer therapies
TheScientificWorldJournal
Scientific reports
Evidence-based complementary and alternative medicine : eCAM
Journal of pharmaceutical and biomedical analysis
Journal of pharmaceutical and biomedical analysis
Zhongguo Zhong yao za zhi = Zhongguo zhongyao zazhi = China journal of Chinese materia me[illegible]
European journal of drug metabolism and pharmacokinetics
Toxicology in vitro : an international journal published in association with BIBRA
Journal of ethnopharmacology
Yao xue xue bao = Acta pharmaceutica Sinica
Journal of separation science
Journal of ethnopharmacology
Journal of chromatographic science
Journal of molecular neuroscience : MN
Toxicological research
Journal of Asian natural products research
Journal of ethnopharmacology
Chinese journal of natural medicines
Journal of chromatographic science
Journal of biomolecular screening
Natural product research
Pharmacological reports : PR
Chinese journal of natural medicines
Journal of separation science

Molecular carcinogenesis
Chinese journal of natural medicines
Chinese journal of natural medicines
Molecules (Basel, Switzerland)
Zhongguo Zhong yao za zhi = Zhongguo zhongyao zazhi = China journal of Chinese materia me
The Analyst
Fitoterapia
Biomedical chromatography : BMC
Xenobiotica; the fate of foreign compounds in biological systems
Rapid communications in mass spectrometry : RCM
Zhong yao cai = Zhongyaocai = Journal of Chinese medicinal materials
Zhongguo Zhong yao za zhi = Zhongguo zhongyao zazhi = China journal of Chinese materia me
Drug testing and analysis
Zhongguo Zhong yao za zhi = Zhongguo zhongyao zazhi = China journal of Chinese materia me
Bioorganic & medicinal chemistry
Journal of ethnopharmacology
Journal of pharmaceutical and biomedical analysis
Evidence-based complementary and alternative medicine : eCAM
Spectrochimica acta. Part A, Molecular and biomolecular spectroscopy
The Journal of organic chemistry
Angewandte Chemie (International ed. in English)
Guang pu xue yu guang pu fen xi = Guang pu
Xenobiotica; the fate of foreign compounds in biological systems
Pakistan journal of pharmaceutical sciences
The Analyst
Natural product research
Analytical chemistry
Journal of separation science
Journal of chromatography. A
Talanta
The journal of physical chemistry. B
Journal of chromatography. B, Analytical technologies in the biomedical and life sciences
Journal of chromatographic science
Journal of pharmaceutical and biomedical analysis
Journal of chromatography. A
Phytotherapy research : PTR
Zhongguo Zhong yao za zhi = Zhongguo zhongyao zazhi = China journal of Chinese materia me
Zhongguo Zhong yao za zhi = Zhongguo zhongyao zazhi = China journal of Chinese materia me
Analytical and bioanalytical chemistry
Evidence-based complementary and alternative medicine : eCAM
Organic & biomolecular chemistry
Journal of ethnopharmacology
Pharmacognosy magazine

Food additives & contaminants. Part A, Chemistry, analysis, control, exposure & risk assessment
Journal of chromatography. B, Analytical technologies in the biomedical and life sciences
Luminescence : the journal of biological and chemical luminescence
Journal of separation science
Zhong yao cai = Zhongyaocai = Journal of Chinese medicinal materials
BMC complementary and alternative medicine
Journal of proteome research
Drug metabolism and disposition: the biological fate of chemicals
Journal of ethnopharmacology
Indian journal of pharmaceutical sciences
BMC complementary and alternative medicine
Journal of separation science
Planta medica
Xenobiotica; the fate of foreign compounds in biological systems
Evidence-based complementary and alternative medicine : eCAM
Materials science & engineering. C, Materials for biological applications
Journal of ethnopharmacology
Scientific reports
Journal of chromatography. A
Zhongguo Zhong yao za zhi = Zhongguo zhongyao zazhi = China journal of Chinese materia me
Journal of pharmaceutical and biomedical analysis
Molecular medicine reports
Journal of chromatographic science
Journal of chromatographic science
Evidence-based complementary and alternative medicine : eCAM
Journal of separation science
Scientific reports
Spectrochimica acta. Part A, Molecular and biomolecular spectroscopy
Zhongguo Zhong xi yi jie he za zhi Zhongguo Zhongxiyi jiehe zazhi = Chinese journal of integrat
The Analyst
Journal of pharmaceutical and biomedical analysis
In vivo (Athens, Greece)
Acta pharmaceutica Sinica. B
Journal of chromatography. B, Analytical technologies in the biomedical and life sciences
Parasitology research
Phytochemical analysis : PCA
Journal of natural products
Journal of ethnopharmacology
Avicenna journal of phytomedicine
Journal of pharmaceutical and biomedical analysis
Planta medica
Life sciences
International immunopharmacology

Spectrochimica acta. Part A, Molecular and biomolecular spectroscopy
Pharmacognosy magazine
Drug development research
Journal of separation science
Analytica chimica acta
Zhongguo Zhong yao za zhi = Zhongguo zhongyao zazhi = China journal of Chinese materia me
Molecular medicine reports
Photodiagnosis and photodynamic therapy
Evidence-based complementary and alternative medicine : eCAM
Zhongguo Zhong yao za zhi = Zhongguo zhongyao zazhi = China journal of Chinese materia me
Biological & pharmaceutical bulletin
Evidence-based complementary and alternative medicine : eCAM
Natural product communications
Evidence-based complementary and alternative medicine : eCAM
Natural product research
Molecules (Basel, Switzerland)
The American journal of Chinese medicine
Photodiagnosis and photodynamic therapy
Journal of global antimicrobial resistance
Pharmaceutical biology
Pharmaceutical biology
BMC complementary and alternative medicine
Molecules (Basel, Switzerland)
International journal of molecular sciences
Journal of microencapsulation
Frontiers in pharmacology
Frontiers in pharmacology
Talanta
Journal of natural medicines
Journal of pharmaceutical and biomedical analysis
The journal of physical chemistry. B
Natural product research
Rapid communications in mass spectrometry : RCM
PloS one
Chinese medicine
Avicenna journal of medical biotechnology
Journal of separation science
Molecules (Basel, Switzerland)
Biochemical and biophysical research communications
European journal of pharmaceutical sciences : official journal of the European Federation for Phar
Biomedical chromatography : BMC
Journal of chromatographic science
Pharmacognosy magazine

International immunopharmacology
International immunopharmacology
Journal of colloid and interface science
European journal of medicinal chemistry
Planta medica
Spectrochimica acta. Part A, Molecular and biomolecular spectroscopy
Biophysical reviews
PeerJ
Journal of microbiology (Seoul, Korea)
Journal of chromatography. B, Analytical technologies in the biomedical and life sciences
Molecules (Basel, Switzerland)
Experimental and therapeutic medicine
Journal of ethnopharmacology
Journal of chromatography. B, Analytical technologies in the biomedical and life sciences
Spectrochimica acta. Part A, Molecular and biomolecular spectroscopy
Analytical chemistry
Zhongguo Zhong yao za zhi = Zhongguo zhongyao zazhi = China journal of Chinese materia me
Zhongguo Zhong yao za zhi = Zhongguo zhongyao zazhi = China journal of Chinese materia me
Natural product research
Zhongguo Zhong yao za zhi = Zhongguo zhongyao zazhi = China journal of Chinese materia me
Zhongguo Zhong yao za zhi = Zhongguo zhongyao zazhi = China journal of Chinese materia me
Zhongguo Zhong yao za zhi = Zhongguo zhongyao zazhi = China journal of Chinese materia me
Journal of separation science
Immunopharmacology and immunotoxicology
Analytical sciences : the international journal of the Japan Society for Analytical Chemistry
Frontiers in pharmacology
Zhongguo Zhong yao za zhi = Zhongguo zhongyao zazhi = China journal of Chinese materia me
Scientific reports
Molecules (Basel, Switzerland)
International journal of immunopathology and pharmacology
Journal of pharmaceutical and biomedical analysis
Human & experimental toxicology
Pharmaceutics
Aging and disease
Journal of pharmaceutical analysis
Journal of pharmaceutical analysis
Journal of pharmaceutical analysis
Journal of ethnopharmacology
Cancer letters
Brain research bulletin
Biochimie
Journal of pharmaceutical and biomedical analysis
Biochimie

Journal of ethnopharmacology
Journal of pharmaceutical and biomedical analysis
Molecules (Basel, Switzerland)
Phytomedicine : international journal of phytotherapy and phytopharmacology
Chinese medicine
Molecules (Basel, Switzerland)
Biomedical chromatography : BMC
Research in pharmaceutical sciences
Biochimica et biophysica acta. General subjects
Acta poloniae pharmaceutica
Journal of medicinal food
Bioorganic & medicinal chemistry
Journal of food and drug analysis
Saudi journal of biological sciences
Zhongguo Zhong yao za zhi = Zhongguo zhongyao zazhi = China journal of Chinese materia me
Biomedical chromatography : BMC
Phytotherapy research : PTR
Physical chemistry chemical physics : PCCP
Journal of chromatography. A
Molecules (Basel, Switzerland)
Yao xue xue bao = Acta pharmaceutica Sinica
Frontiers in plant science
Chinese medicine
Malaria journal
Zhongguo Zhong yao za zhi = Zhongguo zhongyao zazhi = China journal of Chinese materia me
Biomedical chromatography : BMC
Journal of separation science
Molecules (Basel, Switzerland)
Journal of analytical methods in chemistry
MedChemComm
Naunyn-Schmiedeberg's archives of pharmacology
Saudi journal of biological sciences
Beilstein journal of organic chemistry
Zhong yao cai = Zhongyaocai = Journal of Chinese medicinal materials
Journal of pharmaceutical and biomedical analysis
Nanomaterials (Basel, Switzerland)
Pharmacological research
Biological & pharmaceutical bulletin
Analytical chemistry
Se pu = Chinese journal of chromatography
Zhongguo Zhong yao za zhi = Zhongguo zhongyao zazhi = China journal of Chinese materia me
Molecules (Basel, Switzerland)
Zhongguo Zhong yao za zhi = Zhongguo zhongyao zazhi = China journal of Chinese materia me

Molecular immunology
Frontiers in microbiology
Journal of separation science
Journal of separation science
Journal of chromatography. A
Zhongguo Zhong yao za zhi = Zhongguo zhongyao zazhi = China journal of Chinese materia me
Physical chemistry chemical physics : PCCP
Molecules (Basel, Switzerland)
Journal of separation science
Biomedical chromatography : BMC
Phytomedicine : international journal of phytotherapy and phytopharmacology
Journal of chromatographic science
Zhongguo Zhong yao za zhi = Zhongguo zhongyao zazhi = China journal of Chinese materia me
Toxins
Journal of ethnopharmacology
Journal of ethnopharmacology
Biochimie
Biomedical optics express
Cellular and molecular biology (Noisy-le-Grand, France)
Zhongguo Zhong yao za zhi = Zhongguo zhongyao zazhi = China journal of Chinese materia me
Photochemical & photobiological sciences : Official journal of the European Photochemistry Asso
Journal of ethnopharmacology
Journal of pharmaceutical and biomedical analysis
Journal of pharmaceutical and biomedical analysis
Archives of physiology and biochemistry
Current vascular pharmacology
International journal of analytical chemistry
Pesticide biochemistry and physiology
Journal of ethnopharmacology
Bioscience, biotechnology, and biochemistry
Biochemical and biophysical research communications
The Analyst
Phytotherapy research : PTR
Zhongguo Zhong yao za zhi = Zhongguo zhongyao zazhi = China journal of Chinese materia me
Computational biology and chemistry
Journal of cellular biochemistry
Yakugaku zasshi : Journal of the Pharmaceutical Society of Japan
Molecules (Basel, Switzerland)
Journal of pharmaceutical and biomedical analysis
Biomedical chromatography : BMC
Journal of natural medicines
Journal of pharmaceutical and biomedical analysis
Scientific reports

Current topics in medicinal chemistry
Bioscience, biotechnology, and biochemistry
Bioscience, biotechnology, and biochemistry
Molecules (Basel, Switzerland)
BMC chemistry
Talanta
Analytica chimica acta
Journal of ethnopharmacology
The British journal of oral & maxillofacial surgery
Spectrochimica acta. Part A, Molecular and biomolecular spectroscopy
Frontiers in veterinary science
Molecules (Basel, Switzerland)
Scientific reports
Infection and drug resistance
Biochemical pharmacology
Zhongguo Zhong yao za zhi = Zhongguo zhongyao zazhi = China journal of Chinese materia me
Journal of analytical methods in chemistry
Analytical and bioanalytical chemistry
Physical chemistry chemical physics : PCCP
Biofouling
Cells
Malaria journal
Biomedical chromatography : BMC
European journal of pharmacology
Cellular and molecular biology (Noisy-le-Grand, France)
Zhongguo Zhong yao za zhi = Zhongguo zhongyao zazhi = China journal of Chinese materia me
Pharmaceutical biology
Neurochemistry international
Current pharmaceutical biotechnology
Journal of biomolecular structure & dynamics
Journal of biomaterials applications
Journal of separation science
Scientific reports
Frontiers in pharmacology
Journal of animal physiology and animal nutrition
Journal of ethnopharmacology
Life sciences
Biological research
Dose-response : a publication of International Hormesis Society
Journal of ethnopharmacology
Frontiers in pharmacology
Journal of pharmaceutical and biomedical analysis
Current medicinal chemistry

Molecules (Basel, Switzerland)
Journal of natural products
Drug design, development and therapy
ACS omega
ACS omega
Beni-Suef University journal of basic and applied sciences
Phytomedicine : international journal of phytotherapy and phytopharmacology
Journal of pharmaceutical and biomedical analysis
Zhongguo Zhong yao za zhi = Zhongguo zhongyao zazhi = China journal of Chinese materia me
BioMed research international
Journal of molecular liquids
Zhongguo Zhong yao za zhi = Zhongguo zhongyao zazhi = China journal of Chinese materia me
Zhongguo Zhong yao za zhi = Zhongguo zhongyao zazhi = China journal of Chinese materia me
Journal of separation science
BioMed research international
Journal of pharmaceutical and biomedical analysis
Oxidative medicine and cellular longevity
Pharmacological research
Journal of AOAC International
Chemico-biological interactions
Chinese medicine
Biomedical chromatography : BMC
Planta medica
Journal of pharmaceutical and biomedical analysis
Journal of ethnopharmacology
Journal of pharmaceutical and biomedical analysis
Frontiers in pharmacology
Journal of molecular graphics & modelling
Zhongguo Zhong yao za zhi = Zhongguo zhongyao zazhi = China journal of Chinese materia me
Zhongguo Zhong yao za zhi = Zhongguo zhongyao zazhi = China journal of Chinese materia me
Evidence-based complementary and alternative medicine : eCAM
Biomedicine & pharmacotherapy = Biomedecine & pharmacotherapie
Frontiers in cellular neuroscience
Zhongguo Zhong yao za zhi = Zhongguo zhongyao zazhi = China journal of Chinese materia me
Journal of analytical methods in chemistry
Journal of ethnopharmacology
Phytomedicine : international journal of phytotherapy and phytopharmacology
Journal of chromatography. B, Analytical technologies in the biomedical and life sciences
Journal of separation science
Analytical and bioanalytical chemistry
Analytical methods : advancing methods and applications
Oxidative medicine and cellular longevity
Drug design, development and therapy

Biomedical chromatography : BMC
Journal of chromatographic science
Molecules (Basel, Switzerland)
Chinese journal of natural medicines
Current drug metabolism
Phytomedicine : international journal of phytotherapy and phytopharmacology
Frontiers in pharmacology
Frontiers in microbiology
Biophysical chemistry
Pharmacological research
Molecular medicine (Cambridge, Mass.)
Molecules (Basel, Switzerland)
Nanomedicine (London, England)
Phytomedicine : international journal of phytotherapy and phytopharmacology
Journal of chromatography. B, Analytical technologies in the biomedical and life sciences
Pakistan journal of pharmaceutical sciences
Frontiers in pharmacology
Analytica chimica acta
Frontiers in pharmacology
Journal of ethnopharmacology
International immunopharmacology
Frontiers in pharmacology
Drug design, development and therapy
Zhongguo Zhong yao za zhi = Zhongguo zhongyao zazhi = China journal of Chinese materia me
Life (Basel, Switzerland)
Zhongguo Zhong yao za zhi = Zhongguo zhongyao zazhi = China journal of Chinese materia me
The Analyst
Phytomedicine : international journal of phytotherapy and phytopharmacology
Journal of analytical methods in chemistry
Frontiers in pharmacology
Journal of medicinal chemistry
Molecules (Basel, Switzerland)
Biomedical chromatography : BMC
Journal of ethnopharmacology
Molecules (Basel, Switzerland)
PloS one
Combinatorial chemistry & high throughput screening
Journal of separation science
Journal of ethnopharmacology
Zhongguo Zhong yao za zhi = Zhongguo zhongyao zazhi = China journal of Chinese materia me
Analytical methods : advancing methods and applications
Biochemical pharmacology
Biomedicine & pharmacotherapy = Biomedecine & pharmacotherapie

Journal of pharmaceutical and biomedical analysis
Journal of pharmaceutical and biomedical analysis
Disease markers
Drug design, development and therapy
Journal of ethnopharmacology
Frontiers in pharmacology
Evidence-based complementary and alternative medicine : eCAM
Neurochemical research
Frontiers in pharmacology
Frontiers in pharmacology
BMC complementary medicine and therapies
Chinese medicine
Drug development research
Journal of biochemical and molecular toxicology
Insects
Journal of analytical methods in chemistry
BioMed research international
Journal of chromatography. A
Chinese herbal medicines
Current issues in molecular biology
Journal of fungi (Basel, Switzerland)
International journal of molecular sciences
Life (Basel, Switzerland)
Frontiers in pharmacology
Journal of biochemical and molecular toxicology
Journal of ethnopharmacology
Evidence-based complementary and alternative medicine : eCAM
Frontiers in microbiology
Natural product research
Basic & clinical pharmacology & toxicology
Frontiers in pharmacology
European journal of medicinal chemistry
Pharmaceuticals (Basel, Switzerland)
Molecules (Basel, Switzerland)
Bioorganic chemistry
Frontiers in plant science
Chinese medicine
BioMed research international
Molecules (Basel, Switzerland)
Biomedicine & pharmacotherapy = Biomedecine & pharmacotherapie
Frontiers in pharmacology
Life (Basel, Switzerland)
Frontiers in aging neuroscience

Frontiers in bioscience (Elite edition)
Drug design, development and therapy
Journal of ethnopharmacology
Journal of pharmaceutical and biomedical analysis
Frontiers in plant science
Metabolites
International journal of biological macromolecules
Frontiers in nutrition
Computers in biology and medicine
Biotechnology and applied biochemistry
Microbiology spectrum
Tropical medicine and health
Frontiers in pharmacology
Frontiers in neuroscience
Journal of ethnopharmacology
CNS & neurological disorders drug targets
Applied biochemistry and biotechnology
Frontiers in medicine
Journal of ethnopharmacology
Asian journal of pharmaceutical sciences
Zhongguo Zhong yao za zhi = Zhongguo zhongyao zazhi = China journal of Chinese materia me
Heliyon
Journal of biomolecular structure & dynamics
Journal of separation science
Frontiers in pharmacology
Journal of neuroimmunology
Foods (Basel, Switzerland)
Phytomedicine : international journal of phytotherapy and phytopharmacology
Frontiers in chemistry
Drug design, development and therapy
Journal of pharmaceutical and biomedical analysis
Frontiers in pharmacology
Journal of pharmaceutical and biomedical analysis
Biomedicine & pharmacotherapy = Biomedecine & pharmacotherapie
Journal of separation science
Journal of ethnopharmacology
Fitoterapia
Zhongguo Zhong yao za zhi = Zhongguo zhongyao zazhi = China journal of Chinese materia me
Journal of ethnopharmacology
Journal of biochemical and molecular toxicology
BMC veterinary research
Mikrochimica acta
International journal of molecular sciences

Frontiers in immunology
Journal of ethnopharmacology
Journal of ethnopharmacology
Journal of biomolecular structure & dynamics
Journal of ethnopharmacology
Zhongguo Zhong yao za zhi = Zhongguo zhongyao zazhi = China journal of Chinese materia me[illegible]
Zhongguo Zhong yao za zhi = Zhongguo zhongyao zazhi = China journal of Chinese materia me[illegible]
Molecules (Basel, Switzerland)
Chinese journal of integrative medicine
Journal of ethnopharmacology
British poultry science
Phytomedicine : international journal of phytotherapy and phytopharmacology
Zhongguo Zhong yao za zhi = Zhongguo zhongyao zazhi = China journal of Chinese materia me[illegible]
Ecotoxicology and environmental safety
Journal of separation science
Journal of separation science
The Journal of pharmacy and pharmacology
Biomedical chromatography : BMC
Frontiers in pharmacology
Biomedicine & pharmacotherapy = Biomedecine & pharmacotherapie
Journal of ethnopharmacology
European journal of pharmacology
The American journal of Chinese medicine
Natural product research
Molecular neurobiology
ACS omega
Frontiers in plant science
Molecules (Basel, Switzerland)
Journal of ethnopharmacology
Technology and health care : official journal of the European Society for Engineering and Medicin[illegible]
Talanta
Journal of natural medicines
International immunopharmacology
Inflammation
Biological & pharmaceutical bulletin
Brain research
Journal of agricultural and food chemistry
Journal of Asian natural products research
International journal of biological macromolecules
Journal of advanced research
Acta parasitologica
Journal of ethnopharmacology
Molecules (Basel, Switzerland)

Zhongguo Zhong yao za zhi = Zhongguo zhongyao zazhi = China journal of Chinese materia me[illegible]
Journal of ethnopharmacology
Fitoterapia
Journal of molecular graphics & modelling
BMC psychiatry
Journal of pesticide science
Biomedicines
Journal of ethnopharmacology
Current pharmaceutical design
Molecular neurobiology
Drug design, development and therapy
Current topics in medicinal chemistry
Journal of Ayurveda and integrative medicine
Biomedicine & pharmacotherapy = Biomedecine & pharmacotherapie
Viruses
Food chemistry
FASEB journal : official publication of the Federation of American Societies for Experimental Biolo[illegible]
Pharmacological research
Ecotoxicology and environmental safety
Fish & shellfish immunology
FASEB journal : official publication of the Federation of American Societies for Experimental Biolo[illegible]
Nature communications
Chemistry & biodiversity
Marine biotechnology (New York, N.Y.)
ACS omega
The journal of physical chemistry letters
ACS chemical neuroscience
Journal of medicinal chemistry
International immunopharmacology
Mikrochimica acta
Pharmaceuticals (Basel, Switzerland)
International immunopharmacology
Journal of separation science
Journal of chromatography. A
Journal of ethnopharmacology
Frontiers in plant science
Food research international (Ottawa, Ont.)
Journal of ethnopharmacology
Zhongguo Zhong yao za zhi = Zhongguo zhongyao zazhi = China journal of Chinese materia me[illegible]
Journal of ethnopharmacology
Journal of ethnopharmacology
Journal of ethnopharmacology
Scientific reports

The American journal of Chinese medicine
Phytomedicine : international journal of phytotherapy and phytopharmacology
Journal of biomolecular structure & dynamics
Drug development research
Journal of ethnopharmacology
Journal of inflammation research
Current topics in medicinal chemistry
Talanta
Phytomedicine : international journal of phytotherapy and phytopharmacology
Journal of bioenergetics and biomembranes
Journal of ethnopharmacology
Spectrochimica acta. Part A, Molecular and biomolecular spectroscopy
Journal of nanobiotechnology
Molecules (Basel, Switzerland)
Molecules (Basel, Switzerland)
Journal of pharmaceutical and biomedical analysis
ACS omega
Molecules (Basel, Switzerland)

Year	PMID	PDF Link
1977	144834	https://pubmed.ncbi.nlm.nih.gov/144834/
1977	331006	https://pubmed.ncbi.nlm.nih.gov/331006/
1977	895386	https://pubmed.ncbi.nlm.nih.gov/895386/
1976	957909	https://pubmed.ncbi.nlm.nih.gov/957909/
1976	1255408	https://pubmed.ncbi.nlm.nih.gov/1255408/
1991	1934166	https://pubmed.ncbi.nlm.nih.gov/1934166/
1990	2282171	https://pubmed.ncbi.nlm.nih.gov/2282171/
1989	2506894	https://pubmed.ncbi.nlm.nih.gov/2506894/
1989	2747249	https://pubmed.ncbi.nlm.nih.gov/2747249/
1989	2801141	https://pubmed.ncbi.nlm.nih.gov/2801141/
1989	2810448	https://pubmed.ncbi.nlm.nih.gov/2810448/
1989	2816389	https://pubmed.ncbi.nlm.nih.gov/2816389/
1988	3286870	https://pubmed.ncbi.nlm.nih.gov/3286870/
1988	3369286	https://pubmed.ncbi.nlm.nih.gov/3369286/
1988	3402447	https://pubmed.ncbi.nlm.nih.gov/3402447/
1986	3811932	https://pubmed.ncbi.nlm.nih.gov/3811932/
1966	4287923	https://pubmed.ncbi.nlm.nih.gov/4287923/
1972	5018340	https://pubmed.ncbi.nlm.nih.gov/5018340/
1965	5899010	https://pubmed.ncbi.nlm.nih.gov/5899010/
1966	5934972	https://pubmed.ncbi.nlm.nih.gov/5934972/
1983	6191021	https://pubmed.ncbi.nlm.nih.gov/6191021/
1982	7083413	https://pubmed.ncbi.nlm.nih.gov/7083413/
1995	7473059	https://pubmed.ncbi.nlm.nih.gov/7473059/
1994	7533889	https://pubmed.ncbi.nlm.nih.gov/7533889/
1995	7621428	https://pubmed.ncbi.nlm.nih.gov/7621428/
1993	7679763	https://pubmed.ncbi.nlm.nih.gov/7679763/
1994	7951991	https://pubmed.ncbi.nlm.nih.gov/7951991/
1993	8274207	https://pubmed.ncbi.nlm.nih.gov/8274207/
1996	8680588	https://pubmed.ncbi.nlm.nih.gov/8680588/
1996	8720385	https://pubmed.ncbi.nlm.nih.gov/8720385/
1995	8798963	https://pubmed.ncbi.nlm.nih.gov/8798963/
1997	9004542	https://pubmed.ncbi.nlm.nih.gov/9004542/
1997	9225598	https://pubmed.ncbi.nlm.nih.gov/9225598/
1997	9376359	https://pubmed.ncbi.nlm.nih.gov/9376359/
1996	9642405	https://pubmed.ncbi.nlm.nih.gov/9642405/
1998	9802326	https://pubmed.ncbi.nlm.nih.gov/9802326/
1998	9923080	https://pubmed.ncbi.nlm.nih.gov/9923080/
1998	9933992	https://pubmed.ncbi.nlm.nih.gov/9933992/
1999	10069523	https://pubmed.ncbi.nlm.nih.gov/10069523/
1999	10382888	https://pubmed.ncbi.nlm.nih.gov/10382888/
1999	10549584	https://pubmed.ncbi.nlm.nih.gov/10549584/
1999	10549848	https://pubmed.ncbi.nlm.nih.gov/10549848/

1999	10552394	https://pubmed.ncbi.nlm.nih.gov/10552394/
2000	10655659	https://pubmed.ncbi.nlm.nih.gov/10655659/
2000	10670817	https://pubmed.ncbi.nlm.nih.gov/10670817/
2000	10695152	https://pubmed.ncbi.nlm.nih.gov/10695152/
1999	10701913	https://pubmed.ncbi.nlm.nih.gov/10701913/
2000	10761980	https://pubmed.ncbi.nlm.nih.gov/10761980/
2000	10775349	https://pubmed.ncbi.nlm.nih.gov/10775349/
1998	10806810	https://pubmed.ncbi.nlm.nih.gov/10806810/
2000	10812938	https://pubmed.ncbi.nlm.nih.gov/10812938/
2000	10826181	https://pubmed.ncbi.nlm.nih.gov/10826181/
2000	11002412	https://pubmed.ncbi.nlm.nih.gov/11002412/
2000	11014297	https://pubmed.ncbi.nlm.nih.gov/11014297/
2001	11217773	https://pubmed.ncbi.nlm.nih.gov/11217773/
2001	11270727	https://pubmed.ncbi.nlm.nih.gov/11270727/
2001	11473418	https://pubmed.ncbi.nlm.nih.gov/11473418/
2001	11721903	https://pubmed.ncbi.nlm.nih.gov/11721903/
2001	11799783	https://pubmed.ncbi.nlm.nih.gov/11799783/
2001	11833714	https://pubmed.ncbi.nlm.nih.gov/11833714/
2001	11895082	https://pubmed.ncbi.nlm.nih.gov/11895082/
1998	12017002	https://pubmed.ncbi.nlm.nih.gov/12017002/
1999	12205892	https://pubmed.ncbi.nlm.nih.gov/12205892/
1999	12212027	https://pubmed.ncbi.nlm.nih.gov/12212027/
2002	12423366	https://pubmed.ncbi.nlm.nih.gov/12423366/
2002	12428952	https://pubmed.ncbi.nlm.nih.gov/12428952/
2002	12467905	https://pubmed.ncbi.nlm.nih.gov/12467905/
2002	12506995	https://pubmed.ncbi.nlm.nih.gov/12506995/
2000	12512445	https://pubmed.ncbi.nlm.nih.gov/12512445/
2000	12515212	https://pubmed.ncbi.nlm.nih.gov/12515212/
2003	12532460	https://pubmed.ncbi.nlm.nih.gov/12532460/
2003	12590392	https://pubmed.ncbi.nlm.nih.gov/12590392/
2002	12683007	https://pubmed.ncbi.nlm.nih.gov/12683007/
2003	12709895	https://pubmed.ncbi.nlm.nih.gov/12709895/
2003	12713397	https://pubmed.ncbi.nlm.nih.gov/12713397/
2002	12774328	https://pubmed.ncbi.nlm.nih.gov/12774328/
2003	12785057	https://pubmed.ncbi.nlm.nih.gov/12785057/
2003	12852562	https://pubmed.ncbi.nlm.nih.gov/12852562/
2003	12865220	https://pubmed.ncbi.nlm.nih.gov/12865220/
2003	12916091	https://pubmed.ncbi.nlm.nih.gov/12916091/
1959	13629961	https://pubmed.ncbi.nlm.nih.gov/13629961/
1960	13685320	https://pubmed.ncbi.nlm.nih.gov/13685320/
1965	14316670	https://pubmed.ncbi.nlm.nih.gov/14316670/
2003	14587878	https://pubmed.ncbi.nlm.nih.gov/14587878/
2003	14640583	https://pubmed.ncbi.nlm.nih.gov/14640583/

2004	15081299	https://pubmed.ncbi.nlm.nih.gov/15081299/
2004	15099450	https://pubmed.ncbi.nlm.nih.gov/15099450/
2003	15139107	https://pubmed.ncbi.nlm.nih.gov/15139107/
2004	15154983	https://pubmed.ncbi.nlm.nih.gov/15154983/
2004	15281263	https://pubmed.ncbi.nlm.nih.gov/15281263/
2004	15341959	https://pubmed.ncbi.nlm.nih.gov/15341959/
2004	15351046	https://pubmed.ncbi.nlm.nih.gov/15351046/
2004	15476305	https://pubmed.ncbi.nlm.nih.gov/15476305/
2004	15532075	https://pubmed.ncbi.nlm.nih.gov/15532075/
2004	15568768	https://pubmed.ncbi.nlm.nih.gov/15568768/
2004	15595415	https://pubmed.ncbi.nlm.nih.gov/15595415/
2003	15617491	https://pubmed.ncbi.nlm.nih.gov/15617491/
2005	15645506	https://pubmed.ncbi.nlm.nih.gov/15645506/
2004	15663220	https://pubmed.ncbi.nlm.nih.gov/15663220/
2005	15664741	https://pubmed.ncbi.nlm.nih.gov/15664741/
2005	15688637	https://pubmed.ncbi.nlm.nih.gov/15688637/
2005	15698803	https://pubmed.ncbi.nlm.nih.gov/15698803/
2005	15750325	https://pubmed.ncbi.nlm.nih.gov/15750325/
2005	15807993	https://pubmed.ncbi.nlm.nih.gov/15807993/
2005	15907704	https://pubmed.ncbi.nlm.nih.gov/15907704/
2005	15925262	https://pubmed.ncbi.nlm.nih.gov/15925262/
2004	15969129	https://pubmed.ncbi.nlm.nih.gov/15969129/
2005	15974077	https://pubmed.ncbi.nlm.nih.gov/15974077/
2005	16011089	https://pubmed.ncbi.nlm.nih.gov/16011089/
2006	16061339	https://pubmed.ncbi.nlm.nih.gov/16061339/
2005	16167364	https://pubmed.ncbi.nlm.nih.gov/16167364/
2006	16293389	https://pubmed.ncbi.nlm.nih.gov/16293389/
2005	16342690	https://pubmed.ncbi.nlm.nih.gov/16342690/
2005	16468373	https://pubmed.ncbi.nlm.nih.gov/16468373/
2006	16497501	https://pubmed.ncbi.nlm.nih.gov/16497501/
2005	16533018	https://pubmed.ncbi.nlm.nih.gov/16533018/
2006	16549318	https://pubmed.ncbi.nlm.nih.gov/16549318/
2006	16635741	https://pubmed.ncbi.nlm.nih.gov/16635741/
2006	16640839	https://pubmed.ncbi.nlm.nih.gov/16640839/
2006	16678250	https://pubmed.ncbi.nlm.nih.gov/16678250/
2006	16780351	https://pubmed.ncbi.nlm.nih.gov/16780351/
2006	16833085	https://pubmed.ncbi.nlm.nih.gov/16833085/
2006	16856488	https://pubmed.ncbi.nlm.nih.gov/16856488/
2006	17024849	https://pubmed.ncbi.nlm.nih.gov/17024849/
2006	17031605	https://pubmed.ncbi.nlm.nih.gov/17031605/
2006	17059001	https://pubmed.ncbi.nlm.nih.gov/17059001/
2006	17069251	https://pubmed.ncbi.nlm.nih.gov/17069251/
2007	17084577	https://pubmed.ncbi.nlm.nih.gov/17084577/

2007	17127054	https://pubmed.ncbi.nlm.nih.gov/17127054/
2007	17156912	https://pubmed.ncbi.nlm.nih.gov/17156912/
2006	17184122	https://pubmed.ncbi.nlm.nih.gov/17184122/
2006	17225535	https://pubmed.ncbi.nlm.nih.gov/17225535/
1993	17230366	https://pubmed.ncbi.nlm.nih.gov/17230366/
1999	17260290	https://pubmed.ncbi.nlm.nih.gov/17260290/
2007	17311227	https://pubmed.ncbi.nlm.nih.gov/17311227/
2007	17338504	https://pubmed.ncbi.nlm.nih.gov/17338504/
1984	17340259	https://pubmed.ncbi.nlm.nih.gov/17340259/
1982	17396979	https://pubmed.ncbi.nlm.nih.gov/17396979/
1981	17401974	https://pubmed.ncbi.nlm.nih.gov/17401974/
1983	17404995	https://pubmed.ncbi.nlm.nih.gov/17404995/
1983	17404996	https://pubmed.ncbi.nlm.nih.gov/17404996/
2007	17434677	https://pubmed.ncbi.nlm.nih.gov/17434677/
2007	17451725	https://pubmed.ncbi.nlm.nih.gov/17451725/
2007	17512816	https://pubmed.ncbi.nlm.nih.gov/17512816/
2007	17534853	https://pubmed.ncbi.nlm.nih.gov/17534853/
2007	17536728	https://pubmed.ncbi.nlm.nih.gov/17536728/
2007	17537686	https://pubmed.ncbi.nlm.nih.gov/17537686/
2007	17645667	https://pubmed.ncbi.nlm.nih.gov/17645667/
2007	17692848	https://pubmed.ncbi.nlm.nih.gov/17692848/
2008	17804078	https://pubmed.ncbi.nlm.nih.gov/17804078/
2007	17891749	https://pubmed.ncbi.nlm.nih.gov/17891749/
2008	17968851	https://pubmed.ncbi.nlm.nih.gov/17968851/
2007	17994701	https://pubmed.ncbi.nlm.nih.gov/17994701/
2008	18081205	https://pubmed.ncbi.nlm.nih.gov/18081205/
2008	18173721	https://pubmed.ncbi.nlm.nih.gov/18173721/
2008	18204477	https://pubmed.ncbi.nlm.nih.gov/18204477/
2008	18258496	https://pubmed.ncbi.nlm.nih.gov/18258496/
2008	18318017	https://pubmed.ncbi.nlm.nih.gov/18318017/
2008	18387354	https://pubmed.ncbi.nlm.nih.gov/18387354/
2008	18395058	https://pubmed.ncbi.nlm.nih.gov/18395058/
2008	18421749	https://pubmed.ncbi.nlm.nih.gov/18421749/
2008	18425753	https://pubmed.ncbi.nlm.nih.gov/18425753/
2008	18446771	https://pubmed.ncbi.nlm.nih.gov/18446771/
2008	18492807	https://pubmed.ncbi.nlm.nih.gov/18492807/
2008	18618524	https://pubmed.ncbi.nlm.nih.gov/18618524/
2008	18630268	https://pubmed.ncbi.nlm.nih.gov/18630268/
2008	18652357	https://pubmed.ncbi.nlm.nih.gov/18652357/
2008	18671433	https://pubmed.ncbi.nlm.nih.gov/18671433/
2008	18781707	https://pubmed.ncbi.nlm.nih.gov/18781707/
2008	18788977	https://pubmed.ncbi.nlm.nih.gov/18788977/
2008	18804774	https://pubmed.ncbi.nlm.nih.gov/18804774/

2008	19016505	https://pubmed.ncbi.nlm.nih.gov/19016505/
2008	19023536	https://pubmed.ncbi.nlm.nih.gov/19023536/
2009	19064089	https://pubmed.ncbi.nlm.nih.gov/19064089/
2009	19121909	https://pubmed.ncbi.nlm.nih.gov/19121909/
2009	19132839	https://pubmed.ncbi.nlm.nih.gov/19132839/
2009	19148860	https://pubmed.ncbi.nlm.nih.gov/19148860/
2009	19156769	https://pubmed.ncbi.nlm.nih.gov/19156769/
2009	19185133	https://pubmed.ncbi.nlm.nih.gov/19185133/
2008	19216173	https://pubmed.ncbi.nlm.nih.gov/19216173/
2008	19260272	https://pubmed.ncbi.nlm.nih.gov/19260272/
2009	19264437	https://pubmed.ncbi.nlm.nih.gov/19264437/
2009	19286310	https://pubmed.ncbi.nlm.nih.gov/19286310/
2009	19345543	https://pubmed.ncbi.nlm.nih.gov/19345543/
2009	19347864	https://pubmed.ncbi.nlm.nih.gov/19347864/
2009	19387576	https://pubmed.ncbi.nlm.nih.gov/19387576/
2009	19406004	https://pubmed.ncbi.nlm.nih.gov/19406004/
2009	19429334	https://pubmed.ncbi.nlm.nih.gov/19429334/
2009	19472295	https://pubmed.ncbi.nlm.nih.gov/19472295/
2009	19485228	https://pubmed.ncbi.nlm.nih.gov/19485228/
2009	19497345	https://pubmed.ncbi.nlm.nih.gov/19497345/
2009	19532985	https://pubmed.ncbi.nlm.nih.gov/19532985/
2009	19533595	https://pubmed.ncbi.nlm.nih.gov/19533595/
2009	19576980	https://pubmed.ncbi.nlm.nih.gov/19576980/
2009	19639777	https://pubmed.ncbi.nlm.nih.gov/19639777/
2009	19652386	https://pubmed.ncbi.nlm.nih.gov/19652386/
2009	19662580	https://pubmed.ncbi.nlm.nih.gov/19662580/
2009	19673395	https://pubmed.ncbi.nlm.nih.gov/19673395/
2009	19685742	https://pubmed.ncbi.nlm.nih.gov/19685742/
2009	19703289	https://pubmed.ncbi.nlm.nih.gov/19703289/
2009	19715476	https://pubmed.ncbi.nlm.nih.gov/19715476/
2011	19737807	https://pubmed.ncbi.nlm.nih.gov/19737807/
2010	19743526	https://pubmed.ncbi.nlm.nih.gov/19743526/
2009	19747439	https://pubmed.ncbi.nlm.nih.gov/19747439/
2009	19754095	https://pubmed.ncbi.nlm.nih.gov/19754095/
2009	19774594	https://pubmed.ncbi.nlm.nih.gov/19774594/
2010	19818826	https://pubmed.ncbi.nlm.nih.gov/19818826/
2009	19874221	https://pubmed.ncbi.nlm.nih.gov/19874221/
2009	19894534	https://pubmed.ncbi.nlm.nih.gov/19894534/
2009	19899183	https://pubmed.ncbi.nlm.nih.gov/19899183/
2010	19899798	https://pubmed.ncbi.nlm.nih.gov/19899798/
2010	19909759	https://pubmed.ncbi.nlm.nih.gov/19909759/
2010	20005324	https://pubmed.ncbi.nlm.nih.gov/20005324/
2011	20077560	https://pubmed.ncbi.nlm.nih.gov/20077560/

2010	20108175	https://pubmed.ncbi.nlm.nih.gov/20108175/
2010	20138455	https://pubmed.ncbi.nlm.nih.gov/20138455/
2010	20153155	https://pubmed.ncbi.nlm.nih.gov/20153155/
2010	20211233	https://pubmed.ncbi.nlm.nih.gov/20211233/
2011	20234973	https://pubmed.ncbi.nlm.nih.gov/20234973/
2011	20309858	https://pubmed.ncbi.nlm.nih.gov/20309858/
2010	20349713	https://pubmed.ncbi.nlm.nih.gov/20349713/
2010	20349723	https://pubmed.ncbi.nlm.nih.gov/20349723/
2010	20354951	https://pubmed.ncbi.nlm.nih.gov/20354951/
2010	20390750	https://pubmed.ncbi.nlm.nih.gov/20390750/
2010	20410605	https://pubmed.ncbi.nlm.nih.gov/20410605/
2010	20430560	https://pubmed.ncbi.nlm.nih.gov/20430560/
2010	20496087	https://pubmed.ncbi.nlm.nih.gov/20496087/
2010	20541984	https://pubmed.ncbi.nlm.nih.gov/20541984/
2010	20561827	https://pubmed.ncbi.nlm.nih.gov/20561827/
2010	20629387	https://pubmed.ncbi.nlm.nih.gov/20629387/
2010	20643587	https://pubmed.ncbi.nlm.nih.gov/20643587/
2010	20661706	https://pubmed.ncbi.nlm.nih.gov/20661706/
2010	20707195	https://pubmed.ncbi.nlm.nih.gov/20707195/
2010	20712125	https://pubmed.ncbi.nlm.nih.gov/20712125/
2010	20715137	https://pubmed.ncbi.nlm.nih.gov/20715137/
2010	20814427	https://pubmed.ncbi.nlm.nih.gov/20814427/
2010	20828184	https://pubmed.ncbi.nlm.nih.gov/20828184/
2010	20847417	https://pubmed.ncbi.nlm.nih.gov/20847417/
2011	20872909	https://pubmed.ncbi.nlm.nih.gov/20872909/
2010	20930384	https://pubmed.ncbi.nlm.nih.gov/20930384/
2010	20931859	https://pubmed.ncbi.nlm.nih.gov/20931859/
2010	21046728	https://pubmed.ncbi.nlm.nih.gov/21046728/
2010	21046787	https://pubmed.ncbi.nlm.nih.gov/21046787/
2010	21049522	https://pubmed.ncbi.nlm.nih.gov/21049522/
2010	21134573	https://pubmed.ncbi.nlm.nih.gov/21134573/
2011	21154891	https://pubmed.ncbi.nlm.nih.gov/21154891/
2011	21171176	https://pubmed.ncbi.nlm.nih.gov/21171176/
2010	21174768	https://pubmed.ncbi.nlm.nih.gov/21174768/
2011	21182530	https://pubmed.ncbi.nlm.nih.gov/21182530/
2011	21188644	https://pubmed.ncbi.nlm.nih.gov/21188644/
2011	21268250	https://pubmed.ncbi.nlm.nih.gov/21268250/
2011	21281702	https://pubmed.ncbi.nlm.nih.gov/21281702/
2011	21319959	https://pubmed.ncbi.nlm.nih.gov/21319959/
2011	21327819	https://pubmed.ncbi.nlm.nih.gov/21327819/
2011	21401114	https://pubmed.ncbi.nlm.nih.gov/21401114/
2011	21425377	https://pubmed.ncbi.nlm.nih.gov/21425377/
2010	21438408	https://pubmed.ncbi.nlm.nih.gov/21438408/

2011	21441482	https://pubmed.ncbi.nlm.nih.gov/21441482/
2011	21466840	https://pubmed.ncbi.nlm.nih.gov/21466840/
2011	21530777	https://pubmed.ncbi.nlm.nih.gov/21530777/
2011	21536491	https://pubmed.ncbi.nlm.nih.gov/21536491/
2011	21645774	https://pubmed.ncbi.nlm.nih.gov/21645774/
2011	21665451	https://pubmed.ncbi.nlm.nih.gov/21665451/
2011	21671134	https://pubmed.ncbi.nlm.nih.gov/21671134/
2011	21858296	https://pubmed.ncbi.nlm.nih.gov/21858296/
2012	21858757	https://pubmed.ncbi.nlm.nih.gov/21858757/
2011	21890297	https://pubmed.ncbi.nlm.nih.gov/21890297/
2012	21968062	https://pubmed.ncbi.nlm.nih.gov/21968062/
2011	21969791	https://pubmed.ncbi.nlm.nih.gov/21969791/
2011	21976812	https://pubmed.ncbi.nlm.nih.gov/21976812/
2012	22024731	https://pubmed.ncbi.nlm.nih.gov/22024731/
2011	22032134	https://pubmed.ncbi.nlm.nih.gov/22032134/
2011	22066400	https://pubmed.ncbi.nlm.nih.gov/22066400/
2011	22066439	https://pubmed.ncbi.nlm.nih.gov/22066439/
2011	22066451	https://pubmed.ncbi.nlm.nih.gov/22066451/
2011	22097321	https://pubmed.ncbi.nlm.nih.gov/22097321/
2012	22119162	https://pubmed.ncbi.nlm.nih.gov/22119162/
2012	22187343	https://pubmed.ncbi.nlm.nih.gov/22187343/
2012	22220694	https://pubmed.ncbi.nlm.nih.gov/22220694/
2011	22242419	https://pubmed.ncbi.nlm.nih.gov/22242419/
2012	22294283	https://pubmed.ncbi.nlm.nih.gov/22294283/
2012	22313410	https://pubmed.ncbi.nlm.nih.gov/22313410/
2012	22314415	https://pubmed.ncbi.nlm.nih.gov/22314415/
2011	22368871	https://pubmed.ncbi.nlm.nih.gov/22368871/
2012	22387241	https://pubmed.ncbi.nlm.nih.gov/22387241/
2011	22393744	https://pubmed.ncbi.nlm.nih.gov/22393744/
2011	22393745	https://pubmed.ncbi.nlm.nih.gov/22393745/
2012	22393824	https://pubmed.ncbi.nlm.nih.gov/22393824/
2004	22454993	https://pubmed.ncbi.nlm.nih.gov/22454993/
2012	22480315	https://pubmed.ncbi.nlm.nih.gov/22480315/
2012	22503734	https://pubmed.ncbi.nlm.nih.gov/22503734/
2012	22543166	https://pubmed.ncbi.nlm.nih.gov/22543166/
2012	22561681	https://pubmed.ncbi.nlm.nih.gov/22561681/
2012	22651959	https://pubmed.ncbi.nlm.nih.gov/22651959/
2012	22707161	https://pubmed.ncbi.nlm.nih.gov/22707161/
2012	22737858	https://pubmed.ncbi.nlm.nih.gov/22737858/
2012	22769040	https://pubmed.ncbi.nlm.nih.gov/22769040/
2011	22779174	https://pubmed.ncbi.nlm.nih.gov/22779174/
2012	22792803	https://pubmed.ncbi.nlm.nih.gov/22792803/
2013	22855402	https://pubmed.ncbi.nlm.nih.gov/22855402/

2012	22870814	https://pubmed.ncbi.nlm.nih.gov/22870814/
2012	22883103	https://pubmed.ncbi.nlm.nih.gov/22883103/
2012	22944006	https://pubmed.ncbi.nlm.nih.gov/22944006/
2012	22986138	https://pubmed.ncbi.nlm.nih.gov/22986138/
2012	23011273	https://pubmed.ncbi.nlm.nih.gov/23011273/
2012	23019888	https://pubmed.ncbi.nlm.nih.gov/23019888/
2013	23026390	https://pubmed.ncbi.nlm.nih.gov/23026390/
2012	23062825	https://pubmed.ncbi.nlm.nih.gov/23062825/
2013	23065224	https://pubmed.ncbi.nlm.nih.gov/23065224/
2013	23092317	https://pubmed.ncbi.nlm.nih.gov/23092317/
2013	23123224	https://pubmed.ncbi.nlm.nih.gov/23123224/
2013	23127648	https://pubmed.ncbi.nlm.nih.gov/23127648/
2013	23147244	https://pubmed.ncbi.nlm.nih.gov/23147244/
2012	23157257	https://pubmed.ncbi.nlm.nih.gov/23157257/
2013	23159469	https://pubmed.ncbi.nlm.nih.gov/23159469/
2012	23175972	https://pubmed.ncbi.nlm.nih.gov/23175972/
2012	23193393	https://pubmed.ncbi.nlm.nih.gov/23193393/
1994	23195817	https://pubmed.ncbi.nlm.nih.gov/23195817/
2013	23200364	https://pubmed.ncbi.nlm.nih.gov/23200364/
2012	23258986	https://pubmed.ncbi.nlm.nih.gov/23258986/
2013	23290487	https://pubmed.ncbi.nlm.nih.gov/23290487/
2013	23303754	https://pubmed.ncbi.nlm.nih.gov/23303754/
2012	23311151	https://pubmed.ncbi.nlm.nih.gov/23311151/
2013	23322663	https://pubmed.ncbi.nlm.nih.gov/23322663/
2013	23351396	https://pubmed.ncbi.nlm.nih.gov/23351396/
2013	23364614	https://pubmed.ncbi.nlm.nih.gov/23364614/
2014	23403059	https://pubmed.ncbi.nlm.nih.gov/23403059/
2013	23418880	https://pubmed.ncbi.nlm.nih.gov/23418880/
2013	23445637	https://pubmed.ncbi.nlm.nih.gov/23445637/
2012	23451518	https://pubmed.ncbi.nlm.nih.gov/23451518/
2012	23460978	https://pubmed.ncbi.nlm.nih.gov/23460978/
2013	23619018	https://pubmed.ncbi.nlm.nih.gov/23619018/
2013	23628154	https://pubmed.ncbi.nlm.nih.gov/23628154/
2013	23629873	https://pubmed.ncbi.nlm.nih.gov/23629873/
2013	23634170	https://pubmed.ncbi.nlm.nih.gov/23634170/
2013	23668021	https://pubmed.ncbi.nlm.nih.gov/23668021/
2014	23696111	https://pubmed.ncbi.nlm.nih.gov/23696111/
2013	23713286	https://pubmed.ncbi.nlm.nih.gov/23713286/
2013	23747414	https://pubmed.ncbi.nlm.nih.gov/23747414/
2013	23756913	https://pubmed.ncbi.nlm.nih.gov/23756913/
2013	23762131	https://pubmed.ncbi.nlm.nih.gov/23762131/
2013	23816126	https://pubmed.ncbi.nlm.nih.gov/23816126/
2013	23886934	https://pubmed.ncbi.nlm.nih.gov/23886934/

2013	23972976	https://pubmed.ncbi.nlm.nih.gov/23972976/
2014	24001845	https://pubmed.ncbi.nlm.nih.gov/24001845/
2013	24035962	https://pubmed.ncbi.nlm.nih.gov/24035962/
2013	24048516	https://pubmed.ncbi.nlm.nih.gov/24048516/
2014	24105360	https://pubmed.ncbi.nlm.nih.gov/24105360/
2013	24164032	https://pubmed.ncbi.nlm.nih.gov/24164032/
2013	24214833	https://pubmed.ncbi.nlm.nih.gov/24214833/
2013	24215432	https://pubmed.ncbi.nlm.nih.gov/24215432/
2014	24236461	https://pubmed.ncbi.nlm.nih.gov/24236461/
2013	24236982	https://pubmed.ncbi.nlm.nih.gov/24236982/
1986	24248127	https://pubmed.ncbi.nlm.nih.gov/24248127/
2012	24250523	https://pubmed.ncbi.nlm.nih.gov/24250523/
1985	24253887	https://pubmed.ncbi.nlm.nih.gov/24253887/
2014	24269776	https://pubmed.ncbi.nlm.nih.gov/24269776/
2014	24290174	https://pubmed.ncbi.nlm.nih.gov/24290174/
2014	24295909	https://pubmed.ncbi.nlm.nih.gov/24295909/
2014	24316533	https://pubmed.ncbi.nlm.nih.gov/24316533/
2014	24333707	https://pubmed.ncbi.nlm.nih.gov/24333707/
2014	24363282	https://pubmed.ncbi.nlm.nih.gov/24363282/
2013	24379740	https://pubmed.ncbi.nlm.nih.gov/24379740/
2014	24413194	https://pubmed.ncbi.nlm.nih.gov/24413194/
2013	24454495	https://pubmed.ncbi.nlm.nih.gov/24454495/
2014	24469095	https://pubmed.ncbi.nlm.nih.gov/24469095/
2014	24469098	https://pubmed.ncbi.nlm.nih.gov/24469098/
2013	24494560	https://pubmed.ncbi.nlm.nih.gov/24494560/
2015	24577954	https://pubmed.ncbi.nlm.nih.gov/24577954/
2014	24583342	https://pubmed.ncbi.nlm.nih.gov/24583342/
2014	24632014	https://pubmed.ncbi.nlm.nih.gov/24632014/
2013	24689238	https://pubmed.ncbi.nlm.nih.gov/24689238/
2014	24723373	https://pubmed.ncbi.nlm.nih.gov/24723373/
2014	24768769	https://pubmed.ncbi.nlm.nih.gov/24768769/
2015	24771056	https://pubmed.ncbi.nlm.nih.gov/24771056/
2014	24793543	https://pubmed.ncbi.nlm.nih.gov/24793543/
2014	24795799	https://pubmed.ncbi.nlm.nih.gov/24795799/
2014	24797560	https://pubmed.ncbi.nlm.nih.gov/24797560/
2014	24815220	https://pubmed.ncbi.nlm.nih.gov/24815220/
2014	24856764	https://pubmed.ncbi.nlm.nih.gov/24856764/
2015	24872523	https://pubmed.ncbi.nlm.nih.gov/24872523/
2014	24874507	https://pubmed.ncbi.nlm.nih.gov/24874507/
2014	24897106	https://pubmed.ncbi.nlm.nih.gov/24897106/
2014	24905299	https://pubmed.ncbi.nlm.nih.gov/24905299/
2014	24969523	https://pubmed.ncbi.nlm.nih.gov/24969523/
2014	24975280	https://pubmed.ncbi.nlm.nih.gov/24975280/

2015	25043857	https://pubmed.ncbi.nlm.nih.gov/25043857/
2014	25053552	https://pubmed.ncbi.nlm.nih.gov/25053552/
2014	25053555	https://pubmed.ncbi.nlm.nih.gov/25053555/
2014	25093987	https://pubmed.ncbi.nlm.nih.gov/25093987/
2014	25095387	https://pubmed.ncbi.nlm.nih.gov/25095387/
2014	25118336	https://pubmed.ncbi.nlm.nih.gov/25118336/
2014	25128422	https://pubmed.ncbi.nlm.nih.gov/25128422/
2015	25132315	https://pubmed.ncbi.nlm.nih.gov/25132315/
2015	25145883	https://pubmed.ncbi.nlm.nih.gov/25145883/
2014	25156599	https://pubmed.ncbi.nlm.nih.gov/25156599/
2014	25174101	https://pubmed.ncbi.nlm.nih.gov/25174101/
2014	25204150	https://pubmed.ncbi.nlm.nih.gov/25204150/
2015	25209714	https://pubmed.ncbi.nlm.nih.gov/25209714/
2014	25272851	https://pubmed.ncbi.nlm.nih.gov/25272851/
2014	25277281	https://pubmed.ncbi.nlm.nih.gov/25277281/
2014	25278183	https://pubmed.ncbi.nlm.nih.gov/25278183/
2015	25285405	https://pubmed.ncbi.nlm.nih.gov/25285405/
2014	25309613	https://pubmed.ncbi.nlm.nih.gov/25309613/
2015	25315870	https://pubmed.ncbi.nlm.nih.gov/25315870/
2014	25329771	https://pubmed.ncbi.nlm.nih.gov/25329771/
2014	25348493	https://pubmed.ncbi.nlm.nih.gov/25348493/
2014	25358169	https://pubmed.ncbi.nlm.nih.gov/25358169/
2015	25369727	https://pubmed.ncbi.nlm.nih.gov/25369727/
2014	25410064	https://pubmed.ncbi.nlm.nih.gov/25410064/
2015	25412989	https://pubmed.ncbi.nlm.nih.gov/25412989/
2015	25424893	https://pubmed.ncbi.nlm.nih.gov/25424893/
2015	25429435	https://pubmed.ncbi.nlm.nih.gov/25429435/
2015	25447425	https://pubmed.ncbi.nlm.nih.gov/25447425/
2014	25454140	https://pubmed.ncbi.nlm.nih.gov/25454140/
2015	25476359	https://pubmed.ncbi.nlm.nih.gov/25476359/
2015	25526561	https://pubmed.ncbi.nlm.nih.gov/25526561/
2015	25531869	https://pubmed.ncbi.nlm.nih.gov/25531869/
2015	25540292	https://pubmed.ncbi.nlm.nih.gov/25540292/
2015	25543284	https://pubmed.ncbi.nlm.nih.gov/25543284/
2015	25573189	https://pubmed.ncbi.nlm.nih.gov/25573189/
2015	25586479	https://pubmed.ncbi.nlm.nih.gov/25586479/
2014	25612435	https://pubmed.ncbi.nlm.nih.gov/25612435/
2014	25612445	https://pubmed.ncbi.nlm.nih.gov/25612445/
2015	25618762	https://pubmed.ncbi.nlm.nih.gov/25618762/
2015	25632291	https://pubmed.ncbi.nlm.nih.gov/25632291/
2015	25687222	https://pubmed.ncbi.nlm.nih.gov/25687222/
2015	25698248	https://pubmed.ncbi.nlm.nih.gov/25698248/
2015	25709212	https://pubmed.ncbi.nlm.nih.gov/25709212/

2015	25739096	https://pubmed.ncbi.nlm.nih.gov/25739096/
2015	25746576	https://pubmed.ncbi.nlm.nih.gov/25746576/
2015	25829078	https://pubmed.ncbi.nlm.nih.gov/25829078/
2015	25832420	https://pubmed.ncbi.nlm.nih.gov/25832420/
2014	25857157	https://pubmed.ncbi.nlm.nih.gov/25857157/
2015	25880755	https://pubmed.ncbi.nlm.nih.gov/25880755/
2015	25917158	https://pubmed.ncbi.nlm.nih.gov/25917158/
2015	25953522	https://pubmed.ncbi.nlm.nih.gov/25953522/
2015	25956675	https://pubmed.ncbi.nlm.nih.gov/25956675/
2015	26009653	https://pubmed.ncbi.nlm.nih.gov/26009653/
2015	26054937	https://pubmed.ncbi.nlm.nih.gov/26054937/
2015	26110501	https://pubmed.ncbi.nlm.nih.gov/26110501/
2015	26132858	https://pubmed.ncbi.nlm.nih.gov/26132858/
2016	26134304	https://pubmed.ncbi.nlm.nih.gov/26134304/
2015	26229546	https://pubmed.ncbi.nlm.nih.gov/26229546/
2015	26249607	https://pubmed.ncbi.nlm.nih.gov/26249607/
2015	26253577	https://pubmed.ncbi.nlm.nih.gov/26253577/
2015	26268432	https://pubmed.ncbi.nlm.nih.gov/26268432/
2015	26298606	https://pubmed.ncbi.nlm.nih.gov/26298606/
2015	26323154	https://pubmed.ncbi.nlm.nih.gov/26323154/
2016	26340557	https://pubmed.ncbi.nlm.nih.gov/26340557/
2015	26352530	https://pubmed.ncbi.nlm.nih.gov/26352530/
2016	26354947	https://pubmed.ncbi.nlm.nih.gov/26354947/
2016	26363492	https://pubmed.ncbi.nlm.nih.gov/26363492/
2015	26379746	https://pubmed.ncbi.nlm.nih.gov/26379746/
2015	26419924	https://pubmed.ncbi.nlm.nih.gov/26419924/
2015	26423775	https://pubmed.ncbi.nlm.nih.gov/26423775/
2016	26454091	https://pubmed.ncbi.nlm.nih.gov/26454091/
2015	26485915	https://pubmed.ncbi.nlm.nih.gov/26485915/
2015	26517817	https://pubmed.ncbi.nlm.nih.gov/26517817/
2016	26519688	https://pubmed.ncbi.nlm.nih.gov/26519688/
2015	26546523	https://pubmed.ncbi.nlm.nih.gov/26546523/
2014	26579408	https://pubmed.ncbi.nlm.nih.gov/26579408/
2015	26590879	https://pubmed.ncbi.nlm.nih.gov/26590879/
2016	26612497	https://pubmed.ncbi.nlm.nih.gov/26612497/
2016	26627195	https://pubmed.ncbi.nlm.nih.gov/26627195/
2015	26654260	https://pubmed.ncbi.nlm.nih.gov/26654260/
2016	26657578	https://pubmed.ncbi.nlm.nih.gov/26657578/
2015	26693406	https://pubmed.ncbi.nlm.nih.gov/26693406/
2016	26760241	https://pubmed.ncbi.nlm.nih.gov/26760241/
2016	26848702	https://pubmed.ncbi.nlm.nih.gov/26848702/
2016	26876917	https://pubmed.ncbi.nlm.nih.gov/26876917/
2016	26945831	https://pubmed.ncbi.nlm.nih.gov/26945831/

2016	26994318	https://pubmed.ncbi.nlm.nih.gov/26994318/
2016	27018001	https://pubmed.ncbi.nlm.nih.gov/27018001/
2016	27045983	https://pubmed.ncbi.nlm.nih.gov/27045983/
2016	27059766	https://pubmed.ncbi.nlm.nih.gov/27059766/
2016	27086100	https://pubmed.ncbi.nlm.nih.gov/27086100/
2015	27141680	https://pubmed.ncbi.nlm.nih.gov/27141680/
2016	27175745	https://pubmed.ncbi.nlm.nih.gov/27175745/
2016	27181460	https://pubmed.ncbi.nlm.nih.gov/27181460/
2016	27239206	https://pubmed.ncbi.nlm.nih.gov/27239206/
2015	27245034	https://pubmed.ncbi.nlm.nih.gov/27245034/
2016	27298183	https://pubmed.ncbi.nlm.nih.gov/27298183/
2016	27313645	https://pubmed.ncbi.nlm.nih.gov/27313645/
2016	27319140	https://pubmed.ncbi.nlm.nih.gov/27319140/
2016	27340419	https://pubmed.ncbi.nlm.nih.gov/27340419/
2017	27373319	https://pubmed.ncbi.nlm.nih.gov/27373319/
2016	27428938	https://pubmed.ncbi.nlm.nih.gov/27428938/
2016	27430912	https://pubmed.ncbi.nlm.nih.gov/27430912/
2016	27444887	https://pubmed.ncbi.nlm.nih.gov/27444887/
2016	27530832	https://pubmed.ncbi.nlm.nih.gov/27530832/
2016	27558975	https://pubmed.ncbi.nlm.nih.gov/27558975/
2016	27569515	https://pubmed.ncbi.nlm.nih.gov/27569515/
2016	27576439	https://pubmed.ncbi.nlm.nih.gov/27576439/
2016	27589716	https://pubmed.ncbi.nlm.nih.gov/27589716/
2016	27598135	https://pubmed.ncbi.nlm.nih.gov/27598135/
2016	27733080	https://pubmed.ncbi.nlm.nih.gov/27733080/
2016	27774066	https://pubmed.ncbi.nlm.nih.gov/27774066/
2016	27812335	https://pubmed.ncbi.nlm.nih.gov/27812335/
2017	27837804	https://pubmed.ncbi.nlm.nih.gov/27837804/
2017	27858308	https://pubmed.ncbi.nlm.nih.gov/27858308/
2017	27875787	https://pubmed.ncbi.nlm.nih.gov/27875787/
2016	27934093	https://pubmed.ncbi.nlm.nih.gov/27934093/
2017	27976592	https://pubmed.ncbi.nlm.nih.gov/27976592/
2017	28044413	https://pubmed.ncbi.nlm.nih.gov/28044413/
2017	28045966	https://pubmed.ncbi.nlm.nih.gov/28045966/
2017	28053654	https://pubmed.ncbi.nlm.nih.gov/28053654/
2017	28090273	https://pubmed.ncbi.nlm.nih.gov/28090273/
2017	28106326	https://pubmed.ncbi.nlm.nih.gov/28106326/
2017	28146096	https://pubmed.ncbi.nlm.nih.gov/28146096/
2017	28153718	https://pubmed.ncbi.nlm.nih.gov/28153718/
2017	28167234	https://pubmed.ncbi.nlm.nih.gov/28167234/
2017	28178753	https://pubmed.ncbi.nlm.nih.gov/28178753/
2017	28203760	https://pubmed.ncbi.nlm.nih.gov/28203760/
2017	28216883	https://pubmed.ncbi.nlm.nih.gov/28216883/

2017	28236763	https://pubmed.ncbi.nlm.nih.gov/28236763/
2017	28278436	https://pubmed.ncbi.nlm.nih.gov/28278436/
2017	28324720	https://pubmed.ncbi.nlm.nih.gov/28324720/
2017	28359045	https://pubmed.ncbi.nlm.nih.gov/28359045/
2017	28399592	https://pubmed.ncbi.nlm.nih.gov/28399592/
2017	28441540	https://pubmed.ncbi.nlm.nih.gov/28441540/
2015	28510102	https://pubmed.ncbi.nlm.nih.gov/28510102/
2017	28533961	https://pubmed.ncbi.nlm.nih.gov/28533961/
2017	28551874	https://pubmed.ncbi.nlm.nih.gov/28551874/
2017	28570935	https://pubmed.ncbi.nlm.nih.gov/28570935/
2017	28587219	https://pubmed.ncbi.nlm.nih.gov/28587219/
2017	28587391	https://pubmed.ncbi.nlm.nih.gov/28587391/
2017	28647508	https://pubmed.ncbi.nlm.nih.gov/28647508/
2017	28756356	https://pubmed.ncbi.nlm.nih.gov/28756356/
2018	28780486	https://pubmed.ncbi.nlm.nih.gov/28780486/
2017	28780868	https://pubmed.ncbi.nlm.nih.gov/28780868/
2016	28861978	https://pubmed.ncbi.nlm.nih.gov/28861978/
2016	28871677	https://pubmed.ncbi.nlm.nih.gov/28871677/
2018	28871816	https://pubmed.ncbi.nlm.nih.gov/28871816/
2016	28895323	https://pubmed.ncbi.nlm.nih.gov/28895323/
2016	28901114	https://pubmed.ncbi.nlm.nih.gov/28901114/
2016	28936841	https://pubmed.ncbi.nlm.nih.gov/28936841/
2017	28961392	https://pubmed.ncbi.nlm.nih.gov/28961392/
2017	28975862	https://pubmed.ncbi.nlm.nih.gov/28975862/
2017	28993581	https://pubmed.ncbi.nlm.nih.gov/28993581/
2017	29051733	https://pubmed.ncbi.nlm.nih.gov/29051733/
2017	29098836	https://pubmed.ncbi.nlm.nih.gov/29098836/
2017	29138445	https://pubmed.ncbi.nlm.nih.gov/29138445/
2017	29143794	https://pubmed.ncbi.nlm.nih.gov/29143794/
2017	29164959	https://pubmed.ncbi.nlm.nih.gov/29164959/
2018	29202306	https://pubmed.ncbi.nlm.nih.gov/29202306/
2018	29233041	https://pubmed.ncbi.nlm.nih.gov/29233041/
2017	29286316	https://pubmed.ncbi.nlm.nih.gov/29286316/
2017	29344415	https://pubmed.ncbi.nlm.nih.gov/29344415/
2014	29403871	https://pubmed.ncbi.nlm.nih.gov/29403871/
2015	29403947	https://pubmed.ncbi.nlm.nih.gov/29403947/
2017	29404021	https://pubmed.ncbi.nlm.nih.gov/29404021/
2018	29410309	https://pubmed.ncbi.nlm.nih.gov/29410309/
2018	29414301	https://pubmed.ncbi.nlm.nih.gov/29414301/
2018	29427595	https://pubmed.ncbi.nlm.nih.gov/29427595/
2018	29458089	https://pubmed.ncbi.nlm.nih.gov/29458089/
2018	29459236	https://pubmed.ncbi.nlm.nih.gov/29459236/
2018	29474974	https://pubmed.ncbi.nlm.nih.gov/29474974/

2018	29477369	https://pubmed.ncbi.nlm.nih.gov/29477369/
2018	29494887	https://pubmed.ncbi.nlm.nih.gov/29494887/
2018	29498692	https://pubmed.ncbi.nlm.nih.gov/29498692/
2018	29526583	https://pubmed.ncbi.nlm.nih.gov/29526583/
2018	29541156	https://pubmed.ncbi.nlm.nih.gov/29541156/
2018	29561801	https://pubmed.ncbi.nlm.nih.gov/29561801/
2018	29577358	https://pubmed.ncbi.nlm.nih.gov/29577358/
2018	29606969	https://pubmed.ncbi.nlm.nih.gov/29606969/
2018	29634991	https://pubmed.ncbi.nlm.nih.gov/29634991/
2016	29648711	https://pubmed.ncbi.nlm.nih.gov/29648711/
2018	29652553	https://pubmed.ncbi.nlm.nih.gov/29652553/
2018	29680749	https://pubmed.ncbi.nlm.nih.gov/29680749/
2018	29703381	https://pubmed.ncbi.nlm.nih.gov/29703381/
2018	29740248	https://pubmed.ncbi.nlm.nih.gov/29740248/
2018	29751718	https://pubmed.ncbi.nlm.nih.gov/29751718/
2018	29782649	https://pubmed.ncbi.nlm.nih.gov/29782649/
2018	29806105	https://pubmed.ncbi.nlm.nih.gov/29806105/
2018	29850718	https://pubmed.ncbi.nlm.nih.gov/29850718/
2018	29866504	https://pubmed.ncbi.nlm.nih.gov/29866504/
2018	29895786	https://pubmed.ncbi.nlm.nih.gov/29895786/
2016	29906033	https://pubmed.ncbi.nlm.nih.gov/29906033/
2018	29915609	https://pubmed.ncbi.nlm.nih.gov/29915609/
2018	29930696	https://pubmed.ncbi.nlm.nih.gov/29930696/
2018	29941026	https://pubmed.ncbi.nlm.nih.gov/29941026/
2018	29945383	https://pubmed.ncbi.nlm.nih.gov/29945383/
2018	29972688	https://pubmed.ncbi.nlm.nih.gov/29972688/
2018	30035355	https://pubmed.ncbi.nlm.nih.gov/30035355/
2018	30072585	https://pubmed.ncbi.nlm.nih.gov/30072585/
2018	30079260	https://pubmed.ncbi.nlm.nih.gov/30079260/
2017	30108832	https://pubmed.ncbi.nlm.nih.gov/30108832/
2018	30155694	https://pubmed.ncbi.nlm.nih.gov/30155694/
2018	30174521	https://pubmed.ncbi.nlm.nih.gov/30174521/
2018	30202477	https://pubmed.ncbi.nlm.nih.gov/30202477/
2016	30204391	https://pubmed.ncbi.nlm.nih.gov/30204391/
2018	30216792	https://pubmed.ncbi.nlm.nih.gov/30216792/
2018	30227671	https://pubmed.ncbi.nlm.nih.gov/30227671/
2018	30243842	https://pubmed.ncbi.nlm.nih.gov/30243842/
2018	30259882	https://pubmed.ncbi.nlm.nih.gov/30259882/
2018	30345742	https://pubmed.ncbi.nlm.nih.gov/30345742/
2018	30378366	https://pubmed.ncbi.nlm.nih.gov/30378366/
2018	30453712	https://pubmed.ncbi.nlm.nih.gov/30453712/
2018	30486378	https://pubmed.ncbi.nlm.nih.gov/30486378/
2018	30486526	https://pubmed.ncbi.nlm.nih.gov/30486526/

2019	30496979	https://pubmed.ncbi.nlm.nih.gov/30496979/
2018	30559726	https://pubmed.ncbi.nlm.nih.gov/30559726/
2019	30638299	https://pubmed.ncbi.nlm.nih.gov/30638299/
2019	30667161	https://pubmed.ncbi.nlm.nih.gov/30667161/
2019	30712820	https://pubmed.ncbi.nlm.nih.gov/30712820/
2018	30717564	https://pubmed.ncbi.nlm.nih.gov/30717564/
2019	30758003	https://pubmed.ncbi.nlm.nih.gov/30758003/
2019	30781392	https://pubmed.ncbi.nlm.nih.gov/30781392/
2019	30793835	https://pubmed.ncbi.nlm.nih.gov/30793835/
2019	30807650	https://pubmed.ncbi.nlm.nih.gov/30807650/
2020	30902523	https://pubmed.ncbi.nlm.nih.gov/30902523/
2019	30929002	https://pubmed.ncbi.nlm.nih.gov/30929002/
2019	30989895	https://pubmed.ncbi.nlm.nih.gov/30989895/
2019	31027283	https://pubmed.ncbi.nlm.nih.gov/31027283/
2019	31028858	https://pubmed.ncbi.nlm.nih.gov/31028858/
2019	31029761	https://pubmed.ncbi.nlm.nih.gov/31029761/
2019	31051209	https://pubmed.ncbi.nlm.nih.gov/31051209/
2019	31061758	https://pubmed.ncbi.nlm.nih.gov/31061758/
2019	31078155	https://pubmed.ncbi.nlm.nih.gov/31078155/
2019	31090320	https://pubmed.ncbi.nlm.nih.gov/31090320/
2019	31099374	https://pubmed.ncbi.nlm.nih.gov/31099374/
2019	31150794	https://pubmed.ncbi.nlm.nih.gov/31150794/
2019	31153138	https://pubmed.ncbi.nlm.nih.gov/31153138/
2019	31163346	https://pubmed.ncbi.nlm.nih.gov/31163346/
2021	31241354	https://pubmed.ncbi.nlm.nih.gov/31241354/
2020	31291876	https://pubmed.ncbi.nlm.nih.gov/31291876/
2019	31354826	https://pubmed.ncbi.nlm.nih.gov/31354826/
2019	31400784	https://pubmed.ncbi.nlm.nih.gov/31400784/
2019	31446073	https://pubmed.ncbi.nlm.nih.gov/31446073/
2020	31462179	https://pubmed.ncbi.nlm.nih.gov/31462179/
2019	31472967	https://pubmed.ncbi.nlm.nih.gov/31472967/
2019	31482933	https://pubmed.ncbi.nlm.nih.gov/31482933/
2020	31496018	https://pubmed.ncbi.nlm.nih.gov/31496018/
2019	31602938	https://pubmed.ncbi.nlm.nih.gov/31602938/
2019	31606587	https://pubmed.ncbi.nlm.nih.gov/31606587/
2020	31633226	https://pubmed.ncbi.nlm.nih.gov/31633226/
2019	31685744	https://pubmed.ncbi.nlm.nih.gov/31685744/
2019	31752124	https://pubmed.ncbi.nlm.nih.gov/31752124/
2020	31806398	https://pubmed.ncbi.nlm.nih.gov/31806398/
2020	31808576	https://pubmed.ncbi.nlm.nih.gov/31808576/
2020	31894475	https://pubmed.ncbi.nlm.nih.gov/31894475/
2020	31901737	https://pubmed.ncbi.nlm.nih.gov/31901737/
2020	31969583	https://pubmed.ncbi.nlm.nih.gov/31969583/

2020	31976836	https://pubmed.ncbi.nlm.nih.gov/31976836/
2020	31985355	https://pubmed.ncbi.nlm.nih.gov/31985355/
2020	32013749	https://pubmed.ncbi.nlm.nih.gov/32013749/
2020	32054011	https://pubmed.ncbi.nlm.nih.gov/32054011/
2020	32055800	https://pubmed.ncbi.nlm.nih.gov/32055800/
2020	32070614	https://pubmed.ncbi.nlm.nih.gov/32070614/
2020	32106948	https://pubmed.ncbi.nlm.nih.gov/32106948/
2020	32135243	https://pubmed.ncbi.nlm.nih.gov/32135243/
2020	32139146	https://pubmed.ncbi.nlm.nih.gov/32139146/
2020	32151986	https://pubmed.ncbi.nlm.nih.gov/32151986/
2020	32154278	https://pubmed.ncbi.nlm.nih.gov/32154278/
2020	32188132	https://pubmed.ncbi.nlm.nih.gov/32188132/
2020	32193501	https://pubmed.ncbi.nlm.nih.gov/32193501/
2020	32210589	https://pubmed.ncbi.nlm.nih.gov/32210589/
2020	32224138	https://pubmed.ncbi.nlm.nih.gov/32224138/
2020	32237427	https://pubmed.ncbi.nlm.nih.gov/32237427/
2020	32280556	https://pubmed.ncbi.nlm.nih.gov/32280556/
2020	32347364	https://pubmed.ncbi.nlm.nih.gov/32347364/
2020	32400802	https://pubmed.ncbi.nlm.nih.gov/32400802/
2020	32410461	https://pubmed.ncbi.nlm.nih.gov/32410461/
2020	32429356	https://pubmed.ncbi.nlm.nih.gov/32429356/
2020	32513250	https://pubmed.ncbi.nlm.nih.gov/32513250/
2020	32533560	https://pubmed.ncbi.nlm.nih.gov/32533560/
2020	32569674	https://pubmed.ncbi.nlm.nih.gov/32569674/
2020	32583771	https://pubmed.ncbi.nlm.nih.gov/32583771/
2020	32627451	https://pubmed.ncbi.nlm.nih.gov/32627451/
2020	32658562	https://pubmed.ncbi.nlm.nih.gov/32658562/
2020	32711021	https://pubmed.ncbi.nlm.nih.gov/32711021/
2021	32744968	https://pubmed.ncbi.nlm.nih.gov/32744968/
2021	32752947	https://pubmed.ncbi.nlm.nih.gov/32752947/
2021	32799702	https://pubmed.ncbi.nlm.nih.gov/32799702/
2020	32823375	https://pubmed.ncbi.nlm.nih.gov/32823375/
2020	32843671	https://pubmed.ncbi.nlm.nih.gov/32843671/
2020	32848725	https://pubmed.ncbi.nlm.nih.gov/32848725/
2021	32865324	https://pubmed.ncbi.nlm.nih.gov/32865324/
2021	32916235	https://pubmed.ncbi.nlm.nih.gov/32916235/
2020	32926925	https://pubmed.ncbi.nlm.nih.gov/32926925/
2020	32928312	https://pubmed.ncbi.nlm.nih.gov/32928312/
2020	32973415	https://pubmed.ncbi.nlm.nih.gov/32973415/
2021	33022337	https://pubmed.ncbi.nlm.nih.gov/33022337/
2020	33041831	https://pubmed.ncbi.nlm.nih.gov/33041831/
2021	33045623	https://pubmed.ncbi.nlm.nih.gov/33045623/
2021	33138749	https://pubmed.ncbi.nlm.nih.gov/33138749/

2020	33147850	https://pubmed.ncbi.nlm.nih.gov/33147850/
2021	33226219	https://pubmed.ncbi.nlm.nih.gov/33226219/
2020	33235437	https://pubmed.ncbi.nlm.nih.gov/33235437/
2020	33251404	https://pubmed.ncbi.nlm.nih.gov/33251404/
2020	33251466	https://pubmed.ncbi.nlm.nih.gov/33251466/
2021	33457430	https://pubmed.ncbi.nlm.nih.gov/33457430/
2021	33486267	https://pubmed.ncbi.nlm.nih.gov/33486267/
2021	33493743	https://pubmed.ncbi.nlm.nih.gov/33493743/
2020	33496098	https://pubmed.ncbi.nlm.nih.gov/33496098/
2021	33506010	https://pubmed.ncbi.nlm.nih.gov/33506010/
2021	33518856	https://pubmed.ncbi.nlm.nih.gov/33518856/
2021	33645072	https://pubmed.ncbi.nlm.nih.gov/33645072/
2021	33645083	https://pubmed.ncbi.nlm.nih.gov/33645083/
2021	33682338	https://pubmed.ncbi.nlm.nih.gov/33682338/
2021	33748269	https://pubmed.ncbi.nlm.nih.gov/33748269/
2021	33765511	https://pubmed.ncbi.nlm.nih.gov/33765511/
2021	33777318	https://pubmed.ncbi.nlm.nih.gov/33777318/
2021	33872811	https://pubmed.ncbi.nlm.nih.gov/33872811/
2022	33909887	https://pubmed.ncbi.nlm.nih.gov/33909887/
2021	34033838	https://pubmed.ncbi.nlm.nih.gov/34033838/
2021	34059101	https://pubmed.ncbi.nlm.nih.gov/34059101/
2021	34075601	https://pubmed.ncbi.nlm.nih.gov/34075601/
2022	34111890	https://pubmed.ncbi.nlm.nih.gov/34111890/
2021	34118572	https://pubmed.ncbi.nlm.nih.gov/34118572/
2021	34161798	https://pubmed.ncbi.nlm.nih.gov/34161798/
2021	34182412	https://pubmed.ncbi.nlm.nih.gov/34182412/
2021	34211397	https://pubmed.ncbi.nlm.nih.gov/34211397/
2021	34246109	https://pubmed.ncbi.nlm.nih.gov/34246109/
2021	34296568	https://pubmed.ncbi.nlm.nih.gov/34296568/
2021	34296569	https://pubmed.ncbi.nlm.nih.gov/34296569/
2021	34306142	https://pubmed.ncbi.nlm.nih.gov/34306142/
2021	34328091	https://pubmed.ncbi.nlm.nih.gov/34328091/
2021	34366788	https://pubmed.ncbi.nlm.nih.gov/34366788/
2021	34402272	https://pubmed.ncbi.nlm.nih.gov/34402272/
2021	34422435	https://pubmed.ncbi.nlm.nih.gov/34422435/
2022	34428521	https://pubmed.ncbi.nlm.nih.gov/34428521/
2021	34438229	https://pubmed.ncbi.nlm.nih.gov/34438229/
2021	34500403	https://pubmed.ncbi.nlm.nih.gov/34500403/
2021	34510772	https://pubmed.ncbi.nlm.nih.gov/34510772/
2021	34535814	https://pubmed.ncbi.nlm.nih.gov/34535814/
2021	34554148	https://pubmed.ncbi.nlm.nih.gov/34554148/
2021	34567416	https://pubmed.ncbi.nlm.nih.gov/34567416/
2021	34588765	https://pubmed.ncbi.nlm.nih.gov/34588765/

2022	34605575	https://pubmed.ncbi.nlm.nih.gov/34605575/
2022	34664067	https://pubmed.ncbi.nlm.nih.gov/34664067/
2021	34684834	https://pubmed.ncbi.nlm.nih.gov/34684834/
2021	34688466	https://pubmed.ncbi.nlm.nih.gov/34688466/
2021	34719358	https://pubmed.ncbi.nlm.nih.gov/34719358/
2021	34735909	https://pubmed.ncbi.nlm.nih.gov/34735909/
2021	34776976	https://pubmed.ncbi.nlm.nih.gov/34776976/
2021	34777286	https://pubmed.ncbi.nlm.nih.gov/34777286/
2022	34784553	https://pubmed.ncbi.nlm.nih.gov/34784553/
2022	34838694	https://pubmed.ncbi.nlm.nih.gov/34838694/
2021	34875999	https://pubmed.ncbi.nlm.nih.gov/34875999/
2021	34885971	https://pubmed.ncbi.nlm.nih.gov/34885971/
2022	34927467	https://pubmed.ncbi.nlm.nih.gov/34927467/
2022	34942456	https://pubmed.ncbi.nlm.nih.gov/34942456/
2022	35026651	https://pubmed.ncbi.nlm.nih.gov/35026651/
2021	35034886	https://pubmed.ncbi.nlm.nih.gov/35034886/
2021	35046810	https://pubmed.ncbi.nlm.nih.gov/35046810/
2022	35058003	https://pubmed.ncbi.nlm.nih.gov/35058003/
2021	35095505	https://pubmed.ncbi.nlm.nih.gov/35095505/
2022	35150814	https://pubmed.ncbi.nlm.nih.gov/35150814/
2022	35151220	https://pubmed.ncbi.nlm.nih.gov/35151220/
2021	35153752	https://pubmed.ncbi.nlm.nih.gov/35153752/
2022	35173416	https://pubmed.ncbi.nlm.nih.gov/35173416/
2022	35178973	https://pubmed.ncbi.nlm.nih.gov/35178973/
2022	35207560	https://pubmed.ncbi.nlm.nih.gov/35207560/
2022	35285208	https://pubmed.ncbi.nlm.nih.gov/35285208/
2022	35393990	https://pubmed.ncbi.nlm.nih.gov/35393990/
2022	35413645	https://pubmed.ncbi.nlm.nih.gov/35413645/
2022	35441054	https://pubmed.ncbi.nlm.nih.gov/35441054/
2022	35496301	https://pubmed.ncbi.nlm.nih.gov/35496301/
2022	35549263	https://pubmed.ncbi.nlm.nih.gov/35549263/
2022	35566342	https://pubmed.ncbi.nlm.nih.gov/35566342/
2022	35577531	https://pubmed.ncbi.nlm.nih.gov/35577531/
2022	35597409	https://pubmed.ncbi.nlm.nih.gov/35597409/
2022	35630611	https://pubmed.ncbi.nlm.nih.gov/35630611/
2022	35639708	https://pubmed.ncbi.nlm.nih.gov/35639708/
2023	35642108	https://pubmed.ncbi.nlm.nih.gov/35642108/
2022	35671063	https://pubmed.ncbi.nlm.nih.gov/35671063/
2022	35697191	https://pubmed.ncbi.nlm.nih.gov/35697191/
2022	35718516	https://pubmed.ncbi.nlm.nih.gov/35718516/
2022	35748312	https://pubmed.ncbi.nlm.nih.gov/35748312/
2022	35760111	https://pubmed.ncbi.nlm.nih.gov/35760111/
2022	35779424	https://pubmed.ncbi.nlm.nih.gov/35779424/

2022	35780528	https://pubmed.ncbi.nlm.nih.gov/35780528/
2022	35803017	https://pubmed.ncbi.nlm.nih.gov/35803017/
2022	35811658	https://pubmed.ncbi.nlm.nih.gov/35811658/
2022	35812134	https://pubmed.ncbi.nlm.nih.gov/35812134/
2022	35843411	https://pubmed.ncbi.nlm.nih.gov/35843411/
2022	35873589	https://pubmed.ncbi.nlm.nih.gov/35873589/
2022	35873623	https://pubmed.ncbi.nlm.nih.gov/35873623/
2022	35917005	https://pubmed.ncbi.nlm.nih.gov/35917005/
2022	35935824	https://pubmed.ncbi.nlm.nih.gov/35935824/
2022	35935870	https://pubmed.ncbi.nlm.nih.gov/35935870/
2022	35971113	https://pubmed.ncbi.nlm.nih.gov/35971113/
2022	35974396	https://pubmed.ncbi.nlm.nih.gov/35974396/
2022	35976121	https://pubmed.ncbi.nlm.nih.gov/35976121/
2022	35997646	https://pubmed.ncbi.nlm.nih.gov/35997646/
2022	36005300	https://pubmed.ncbi.nlm.nih.gov/36005300/
2022	36059925	https://pubmed.ncbi.nlm.nih.gov/36059925/
2022	36060126	https://pubmed.ncbi.nlm.nih.gov/36060126/
2022	36063777	https://pubmed.ncbi.nlm.nih.gov/36063777/
2020	36117558	https://pubmed.ncbi.nlm.nih.gov/36117558/
2022	36135206	https://pubmed.ncbi.nlm.nih.gov/36135206/
2022	36135681	https://pubmed.ncbi.nlm.nih.gov/36135681/
2022	36142236	https://pubmed.ncbi.nlm.nih.gov/36142236/
2022	36143445	https://pubmed.ncbi.nlm.nih.gov/36143445/
2022	36160407	https://pubmed.ncbi.nlm.nih.gov/36160407/
2023	36169195	https://pubmed.ncbi.nlm.nih.gov/36169195/
2023	36198377	https://pubmed.ncbi.nlm.nih.gov/36198377/
2022	36204127	https://pubmed.ncbi.nlm.nih.gov/36204127/
2022	36212891	https://pubmed.ncbi.nlm.nih.gov/36212891/
2023	36260052	https://pubmed.ncbi.nlm.nih.gov/36260052/
2023	36303263	https://pubmed.ncbi.nlm.nih.gov/36303263/
2022	36304144	https://pubmed.ncbi.nlm.nih.gov/36304144/
2023	36347091	https://pubmed.ncbi.nlm.nih.gov/36347091/
2022	36355489	https://pubmed.ncbi.nlm.nih.gov/36355489/
2022	36364339	https://pubmed.ncbi.nlm.nih.gov/36364339/
2023	36371822	https://pubmed.ncbi.nlm.nih.gov/36371822/
2022	36388527	https://pubmed.ncbi.nlm.nih.gov/36388527/
2022	36434688	https://pubmed.ncbi.nlm.nih.gov/36434688/
2022	36452059	https://pubmed.ncbi.nlm.nih.gov/36452059/
2022	36500340	https://pubmed.ncbi.nlm.nih.gov/36500340/
2023	36502756	https://pubmed.ncbi.nlm.nih.gov/36502756/
2022	36532769	https://pubmed.ncbi.nlm.nih.gov/36532769/
2022	36556479	https://pubmed.ncbi.nlm.nih.gov/36556479/
2022	36570530	https://pubmed.ncbi.nlm.nih.gov/36570530/

2022	36575849	https://pubmed.ncbi.nlm.nih.gov/36575849/
2022	36578822	https://pubmed.ncbi.nlm.nih.gov/36578822/
2023	36581161	https://pubmed.ncbi.nlm.nih.gov/36581161/
2023	36603394	https://pubmed.ncbi.nlm.nih.gov/36603394/
2022	36618650	https://pubmed.ncbi.nlm.nih.gov/36618650/
2022	36676934	https://pubmed.ncbi.nlm.nih.gov/36676934/
2023	36682666	https://pubmed.ncbi.nlm.nih.gov/36682666/
2022	36698459	https://pubmed.ncbi.nlm.nih.gov/36698459/
2023	36731363	https://pubmed.ncbi.nlm.nih.gov/36731363/
2023	36806191	https://pubmed.ncbi.nlm.nih.gov/36806191/
2023	36809123	https://pubmed.ncbi.nlm.nih.gov/36809123/
2023	36859380	https://pubmed.ncbi.nlm.nih.gov/36859380/
2023	36874034	https://pubmed.ncbi.nlm.nih.gov/36874034/
2023	36875667	https://pubmed.ncbi.nlm.nih.gov/36875667/
2023	37028611	https://pubmed.ncbi.nlm.nih.gov/37028611/
2024	37073143	https://pubmed.ncbi.nlm.nih.gov/37073143/
2024	37099127	https://pubmed.ncbi.nlm.nih.gov/37099127/
2023	37153081	https://pubmed.ncbi.nlm.nih.gov/37153081/
2023	37211189	https://pubmed.ncbi.nlm.nih.gov/37211189/
2023	37274923	https://pubmed.ncbi.nlm.nih.gov/37274923/
2023	37282971	https://pubmed.ncbi.nlm.nih.gov/37282971/
2023	37305452	https://pubmed.ncbi.nlm.nih.gov/37305452/
2024	37315995	https://pubmed.ncbi.nlm.nih.gov/37315995/
2023	37339806	https://pubmed.ncbi.nlm.nih.gov/37339806/
2023	37351515	https://pubmed.ncbi.nlm.nih.gov/37351515/
2023	37413943	https://pubmed.ncbi.nlm.nih.gov/37413943/
2022	37430999	https://pubmed.ncbi.nlm.nih.gov/37430999/
2023	37516057	https://pubmed.ncbi.nlm.nih.gov/37516057/
2023	37583566	https://pubmed.ncbi.nlm.nih.gov/37583566/
2023	37609432	https://pubmed.ncbi.nlm.nih.gov/37609432/
2023	37633162	https://pubmed.ncbi.nlm.nih.gov/37633162/
2023	37637408	https://pubmed.ncbi.nlm.nih.gov/37637408/
2023	37708763	https://pubmed.ncbi.nlm.nih.gov/37708763/
2023	37729733	https://pubmed.ncbi.nlm.nih.gov/37729733/
2023	37737621	https://pubmed.ncbi.nlm.nih.gov/37737621/
2024	37778519	https://pubmed.ncbi.nlm.nih.gov/37778519/
2023	37797794	https://pubmed.ncbi.nlm.nih.gov/37797794/
2023	37802806	https://pubmed.ncbi.nlm.nih.gov/37802806/
2024	37813288	https://pubmed.ncbi.nlm.nih.gov/37813288/
2024	37815058	https://pubmed.ncbi.nlm.nih.gov/37815058/
2023	37880653	https://pubmed.ncbi.nlm.nih.gov/37880653/
2023	37999777	https://pubmed.ncbi.nlm.nih.gov/37999777/
2023	38003731	https://pubmed.ncbi.nlm.nih.gov/38003731/

2023	38035098	https://pubmed.ncbi.nlm.nih.gov/38035098/
2024	38072292	https://pubmed.ncbi.nlm.nih.gov/38072292/
2024	38072294	https://pubmed.ncbi.nlm.nih.gov/38072294/
2025	38100552	https://pubmed.ncbi.nlm.nih.gov/38100552/
2024	38104873	https://pubmed.ncbi.nlm.nih.gov/38104873/
2023	38114149	https://pubmed.ncbi.nlm.nih.gov/38114149/
2023	38114174	https://pubmed.ncbi.nlm.nih.gov/38114174/
2023	38138445	https://pubmed.ncbi.nlm.nih.gov/38138445/
2024	38157118	https://pubmed.ncbi.nlm.nih.gov/38157118/
2024	38160864	https://pubmed.ncbi.nlm.nih.gov/38160864/
2024	38166582	https://pubmed.ncbi.nlm.nih.gov/38166582/
2024	38181529	https://pubmed.ncbi.nlm.nih.gov/38181529/
2023	38212008	https://pubmed.ncbi.nlm.nih.gov/38212008/
2024	38218103	https://pubmed.ncbi.nlm.nih.gov/38218103/
2024	38234032	https://pubmed.ncbi.nlm.nih.gov/38234032/
2024	38234033	https://pubmed.ncbi.nlm.nih.gov/38234033/
2024	38280221	https://pubmed.ncbi.nlm.nih.gov/38280221/
2024	38317273	https://pubmed.ncbi.nlm.nih.gov/38317273/
2024	38318139	https://pubmed.ncbi.nlm.nih.gov/38318139/
2024	38325264	https://pubmed.ncbi.nlm.nih.gov/38325264/
2024	38341112	https://pubmed.ncbi.nlm.nih.gov/38341112/
2024	38350592	https://pubmed.ncbi.nlm.nih.gov/38350592/
2024	38351702	https://pubmed.ncbi.nlm.nih.gov/38351702/
2024	38353142	https://pubmed.ncbi.nlm.nih.gov/38353142/
2024	38401045	https://pubmed.ncbi.nlm.nih.gov/38401045/
2024	38434828	https://pubmed.ncbi.nlm.nih.gov/38434828/
2024	38450408	https://pubmed.ncbi.nlm.nih.gov/38450408/
2024	38474561	https://pubmed.ncbi.nlm.nih.gov/38474561/
2024	38499258	https://pubmed.ncbi.nlm.nih.gov/38499258/
2024	38517810	https://pubmed.ncbi.nlm.nih.gov/38517810/
2024	38537350	https://pubmed.ncbi.nlm.nih.gov/38537350/
2024	38573419	https://pubmed.ncbi.nlm.nih.gov/38573419/
2024	38579565	https://pubmed.ncbi.nlm.nih.gov/38579565/
2024	38598115	https://pubmed.ncbi.nlm.nih.gov/38598115/
2024	38599826	https://pubmed.ncbi.nlm.nih.gov/38599826/
2024	38609032	https://pubmed.ncbi.nlm.nih.gov/38609032/
2024	38619332	https://pubmed.ncbi.nlm.nih.gov/38619332/
2024	38619479	https://pubmed.ncbi.nlm.nih.gov/38619479/
2024	38643915	https://pubmed.ncbi.nlm.nih.gov/38643915/
2025	38677546	https://pubmed.ncbi.nlm.nih.gov/38677546/
2024	38705947	https://pubmed.ncbi.nlm.nih.gov/38705947/
2024	38789094	https://pubmed.ncbi.nlm.nih.gov/38789094/
2024	38792233	https://pubmed.ncbi.nlm.nih.gov/38792233/

2024	38812197	https://pubmed.ncbi.nlm.nih.gov/38812197/
2024	38823658	https://pubmed.ncbi.nlm.nih.gov/38823658/
2024	38838823	https://pubmed.ncbi.nlm.nih.gov/38838823/
2024	38852429	https://pubmed.ncbi.nlm.nih.gov/38852429/
2024	38867159	https://pubmed.ncbi.nlm.nih.gov/38867159/
2024	38882708	https://pubmed.ncbi.nlm.nih.gov/38882708/
2024	38927546	https://pubmed.ncbi.nlm.nih.gov/38927546/
2024	39038504	https://pubmed.ncbi.nlm.nih.gov/39038504/
2024	39076092	https://pubmed.ncbi.nlm.nih.gov/39076092/
2025	39096445	https://pubmed.ncbi.nlm.nih.gov/39096445/
2024	39109327	https://pubmed.ncbi.nlm.nih.gov/39109327/
2024	39136504	https://pubmed.ncbi.nlm.nih.gov/39136504/
2024	39181067	https://pubmed.ncbi.nlm.nih.gov/39181067/
2024	39182321	https://pubmed.ncbi.nlm.nih.gov/39182321/
2024	39205203	https://pubmed.ncbi.nlm.nih.gov/39205203/
2025	39241417	https://pubmed.ncbi.nlm.nih.gov/39241417/
2024	39287361	https://pubmed.ncbi.nlm.nih.gov/39287361/
2024	39293583	https://pubmed.ncbi.nlm.nih.gov/39293583/
2024	39332194	https://pubmed.ncbi.nlm.nih.gov/39332194/
2024	39332654	https://pubmed.ncbi.nlm.nih.gov/39332654/
2024	39373933	https://pubmed.ncbi.nlm.nih.gov/39373933/
2024	39384562	https://pubmed.ncbi.nlm.nih.gov/39384562/
2025	39387347	https://pubmed.ncbi.nlm.nih.gov/39387347/
2024	39388028	https://pubmed.ncbi.nlm.nih.gov/39388028/
2024	39398137	https://pubmed.ncbi.nlm.nih.gov/39398137/
2024	39401411	https://pubmed.ncbi.nlm.nih.gov/39401411/
2024	39404232	https://pubmed.ncbi.nlm.nih.gov/39404232/
2024	39420825	https://pubmed.ncbi.nlm.nih.gov/39420825/
2024	39423661	https://pubmed.ncbi.nlm.nih.gov/39423661/
2024	39438355	https://pubmed.ncbi.nlm.nih.gov/39438355/
2024	39458972	https://pubmed.ncbi.nlm.nih.gov/39458972/
2024	39536490	https://pubmed.ncbi.nlm.nih.gov/39536490/
2024	39540876	https://pubmed.ncbi.nlm.nih.gov/39540876/
2025	39566289	https://pubmed.ncbi.nlm.nih.gov/39566289/
2025	39566861	https://pubmed.ncbi.nlm.nih.gov/39566861/
2024	39574447	https://pubmed.ncbi.nlm.nih.gov/39574447/
2024	39593335	https://pubmed.ncbi.nlm.nih.gov/39593335/
2025	39701220	https://pubmed.ncbi.nlm.nih.gov/39701220/
2024	39701727	https://pubmed.ncbi.nlm.nih.gov/39701727/
2025	39710160	https://pubmed.ncbi.nlm.nih.gov/39710160/
2025	39733801	https://pubmed.ncbi.nlm.nih.gov/39733801/
2025	39746410	https://pubmed.ncbi.nlm.nih.gov/39746410/
2025	39870866	https://pubmed.ncbi.nlm.nih.gov/39870866/

2025	39880666	https://pubmed.ncbi.nlm.nih.gov/39880666/
2025	39933468	https://pubmed.ncbi.nlm.nih.gov/39933468/
2025	40053458	https://pubmed.ncbi.nlm.nih.gov/40053458/
2025	40079275	https://pubmed.ncbi.nlm.nih.gov/40079275/
2025	40089197	https://pubmed.ncbi.nlm.nih.gov/40089197/
2025	40093952	https://pubmed.ncbi.nlm.nih.gov/40093952/
2025	40129141	https://pubmed.ncbi.nlm.nih.gov/40129141/
2025	40132407	https://pubmed.ncbi.nlm.nih.gov/40132407/
2025	40138773	https://pubmed.ncbi.nlm.nih.gov/40138773/
2025	40172736	https://pubmed.ncbi.nlm.nih.gov/40172736/
2025	40188897	https://pubmed.ncbi.nlm.nih.gov/40188897/
2025	40215854	https://pubmed.ncbi.nlm.nih.gov/40215854/
2025	40275381	https://pubmed.ncbi.nlm.nih.gov/40275381/
2025	40286019	https://pubmed.ncbi.nlm.nih.gov/40286019/
2025	40333914	https://pubmed.ncbi.nlm.nih.gov/40333914/
2025	40381250	https://pubmed.ncbi.nlm.nih.gov/40381250/
2025	40415848	https://pubmed.ncbi.nlm.nih.gov/40415848/
2025	40430300	https://pubmed.ncbi.nlm.nih.gov/40430300/
